# Direct transdifferentiation of tumorigenic melanoma cells induces tumor cell reversion

**DOI:** 10.1038/s41419-025-07863-y

**Published:** 2025-07-25

**Authors:** Yiman Wang, Ke Liu, Yuxin Zhang, Daniel Novak, Aniello Federico, Cai Xu, Sandra Horschitz, Marlene Vierthaler, Qian Sun, Nina Wang, Juliane Poelchen, Tamara Steinfass, Laura Hüser, Moritz Mall, Viktor Umansky, Jochen Utikal

**Affiliations:** 1 Skin Cancer Unit, German Cancer Research Center (DKFZ), Heidelberg, Baden-Württemberg Germany; 2 Department of Dermatology, Venereology and Allergology, University Medical Center Mannheim, Ruprecht-Karl University of Heidelberg, Mannheim, Baden-Württemberg Germany; 3 DKFZ-Hector Cancer Institute at the University Medical Centre Mannheim, Mannheim, Germany; 4 Hopp Children’s Cancer Center Heidelberg (KiTZ), Heidelberg, Germany; 5 Division of Pediatric Neuro-Oncology, German Cancer Research Center (DKFZ), German Cancer Consortium (DKTK), Heidelberg, Germany; 6 Radiooncology/Radiobiology Department, German Cancer Research Center (DKFZ), Heidelberg, Baden-Württemberg Germany; 7 Department of Translational Brain Research, Central Institute of Mental Health (ZI), University of Heidelberg/Medical Faculty, Mannheim, Germany; 8 HITBR Hector Institute for Translational Brain Research gGmbH, Mannheim, Germany; 9 German Cancer Research Center (DKFZ), Heidelberg, Germany; 10 Division of Biochemistry and Molecular Biology, Johns Hopkins Bloomberg School of Public Health, Baltimore, MD USA; 11 Cell Fate Engineering and Disease Modeling Group, German Cancer Research Center (DKFZ) and DKFZ-ZMBH Alliance, Heidelberg, Germany; 12 HITBR Hector Institute for Translational Brain Research GmbH, Heidelberg, Germany; 13 Central Institute of Mental Health, Medical Faculty Mannheim, Heidelberg University, Mannheim, Germany; 14 Mannheim Institute for Innate Immunoscience (MI3), Medical Faculty Mannheim, University of Heidelberg, Mannheim, Germany

**Keywords:** Cancer, Preclinical research

## Abstract

Melanoma is an aggressive skin cancer and highly lethal at advanced stages due to its high tumorigenicity and metastatic capacity. Changing the phenotype of cancer cells from one lineage to another, a process called transdifferentiation, leads to tumor cell reversion, which goes along with a drastic reduction of their tumorigenicity. Via ectopic overexpression of four neuronal transcription factors, we transdifferentiated melanoma cells into neuron-like cells expressing neuronal markers and showing a neuron-like morphology. Moreover, the tumorigenic and metastatic potential of transdifferentiated cells in vitro and in vivo was significantly reduced. Transdifferentiated cells were also more sensitive to radiotherapy compared with their parental counterparts. We conclude that transdifferentiation of cancer cells into terminally differentiated neuron-like cells might represent a prospective new therapeutic approach for the treatment of melanoma.

## Introduction

Malignant transformation of melanocytes leads to the development of melanoma, a highly aggressive skin cancer that is responsible for the majority of skin cancer-related deaths despite accounting for only about 4% of all skin cancer cases [1]. There is evidence that changing the lineage of origin of a cancer cell leads to a drastic reduction of its tumorigenic potential. Various groups could directly transdifferentiate one somatic cell type into another by overexpressing lineage-specific transcription factors [2]. Soon after, it was demonstrated that cancer cells are also susceptible to transdifferentiation. Rapino and colleagues reported that they could convert B lymphoma and leukemia cells into macrophages just by ectopically overexpressing the transcription factor C/EBPa. Interestingly, the transdifferentiated cells lost their tumorigenic potential [3]. Likewise, the transdifferentiation of squamous cell carcinoma cells to melanocyte-like cells goes along with a significant reduction of tumorigenic capacity, confirming that the tumor phenotype is associated with a specific differentiation lineage [4]. Moreover, converting melanoma and other tumor cells into a pluripotent-like state and subsequently differentiating them completely abrogated the tumorigenic potential of these cells [5–7]. These examples confirm that tumor reversion, which is defined as the change of phenotype from a malignant cancer cell to a benign, differentiated somatic cell, can be achieved by changing the lineage of origin of cancer cells [8]. In this study, we investigated whether melanoma cells can be transdifferentiated into neurons. Neurons were chosen since terminally differentiated neurons are postmitotic. For this purpose, we ectopically overexpressed the neuron-specific transcription factors ASCL1, BRN2, MYT1L, and NEUROD1 in melanoma cells. The overexpression of these four factors has proven to be sufficient to promote the transdifferentiation of fibroblasts toward neurons [9, 10]. We could show that melanoma cells indeed could be transdifferentiated into neuron-like cells that expressed neuronal markers and showed a neuron-like morphology. Moreover, the transdifferentiated cells had a significantly reduced tumorigenic potential and were more sensitive to radiotherapy compared to the parental melanoma cells.

## Materials and methods

### Plasmids

pLU-EF1aL-rtTA3-iCherry was obtained from Meenhard Herlyn from the Wistar Institute in Philadelphia, USA. TetO-*Ascl1*-puro was a gift from Marius Wernig (Addgene plasmid # 97329; http://n2t.net/addgene:97329; RRID: Addgene_97329) [11]. The ORFs of murine *Brn2*, *Myt1L*, and *NeuroD1* were cloned into our own doxycycline-inducible lentiviral vector system by GenScript (Hong Kong).

### Cell culture

All parental melanoma cell lines HT144 (RRID:CVCL_0318), A375 (RRID:CVCL_0132), SKMel103 (RRID:CVCL_6069), and MA1 [12] were cultivated in DMEM (Gibco, Life Technologies) supplemented with 10% FCS (Biochrom), 1% penicillin (100 units/mL, Sigma-Aldrich) and streptomycin (100 μg/mL, Sigma-Aldrich) (pen/strep), 1% (v/v) of non-essential amino acid solution (NEAA,10 mM, Sigma-Aldrich) and 0.1% (v/v) β-mercaptoethanol (Gibco, Life Technologies). All melanoma cell lines were cultured in a humidified incubator with 5% CO_2_ at 37 °C. Cells were passaged using a 21 mM trypsin solution (Sigma-Aldrich) when reaching 70-90% confluency. If not otherwise specified, transdifferentiated cells were also cultured in the same medium as the parental cells.

### Production of lentiviral particles and transduction

HEK293T cells (RRID:CVCL_0063) were used for lentiviral particle production. X-tremeGENE® transfection reagent (Roche) was used according to the manufacturer’s instructions to transfect the HEK cells. One milliliter of filtered supernatant containing viral particles was used for transducing melanoma cells in one well of a 6-well plate. Forty-eight hours after transduction, the cells were washed with PBS and fresh medium without virus.

### Generation of subclones harboring all four neuron-specific expression constructs

**Fig. 1:**
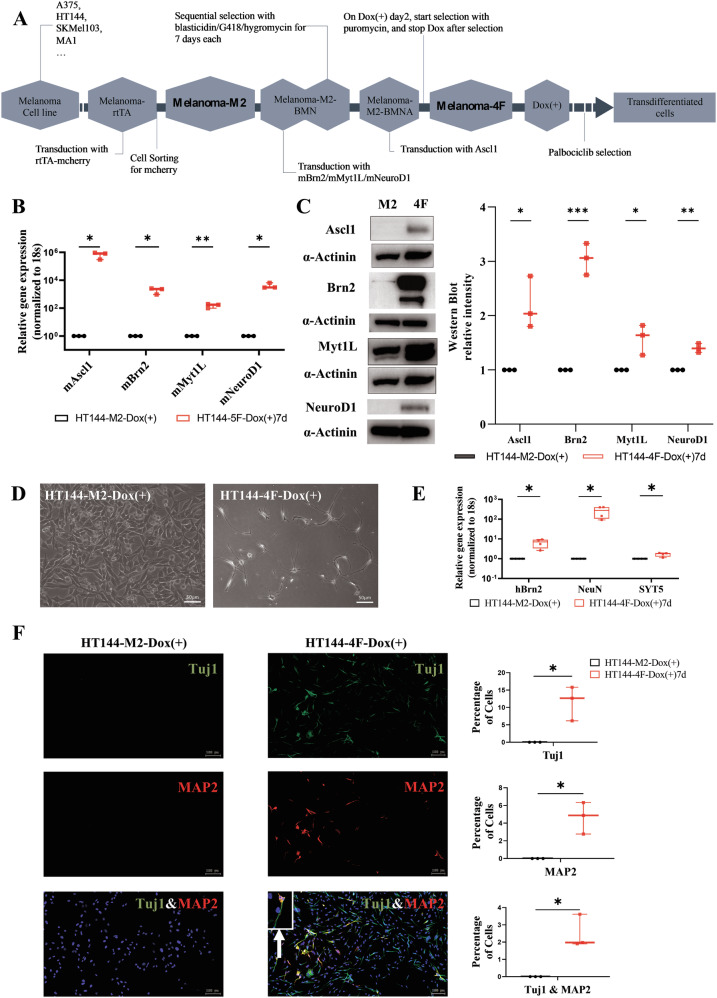
Melanoma cells transdifferentiate into neuron-like cells. **A** Schematic overview of the 4-factor (4F) tumor cell transdifferentiation procedure. **B**, **C** Verification of the ectopic overexpression of the factors used for the transdifferentiation of cells into neurons. **D** Comparison of the morphologies of transdifferentiating and parental melanoma cells. After 14 days of treatment with doxycycline and enrichment by palbociclib treatment, neuron-like cells with round somata and long processes were visible. **E** The expression of neuronal markers was elevated at the RNA level after treatment with doxycycline for 7 days. **F** Expression of neuronal markers could also be demonstrated at the protein level via immunostaining 7 days after induction with doxycycline.

HT144, A375, SKMel103, and MA1 melanoma cells were transduced with pLU-EF1aL-rtTA3-iCherry. The cells were then selected for mCherry fluorescence using a cell sorter, BD FACSAria, from the Flow Cytometry Core Facility of the DKFZ. Successfully transduced cells were hereafter termed *Melanoma-M2*. Then the cells were sequentially transduced with the expression constructs for the factors BRN2, MYT1L, and NEUROD1 and afterwards treated with 3 different antibiotics (blasticidin, hygromycin, and G418) in order to select for cells transduced with the respective constructs. The selected cells were transduced with an expression construct coding for ASCL1 and, after induction with doxycycline (1 µg/ml), subjected to selection with puromycin. *Melanoma-M2* cells infected with and selected for all four transcription factors were named *Melanoma-4F* (Fig. 1A).

### Transdifferentiation of melanoma cells

The melanoma cells that were successfully selected for all four factors were used for the transdifferentiation experiments. The cells were grown in DMEM supplemented with 10% FCS, 1% pen/strep, 1% (v/v) NEAA, 0.1% (v/v) β-mercaptoethanol, and doxycycline (1 µg/ml). Doxycycline induced the expression of the transdifferentiation factors ASCL1, BRN2, MYT1L, and NEUROD1. The medium was changed every 2–3 days. After a minimum of 4 days, cells were used for functional studies or molecular analyses.

### Quantitative real-time PCR

Total RNA was extracted using the RNeasy Mini Kit (QIAGEN) according to the manufacturer’s instructions. A NanoDrop ND-1000 spectrophotometer was used to measure the quality and quantity of RNA samples. Five hundred nanograms of RNA were used to synthesize cDNA with the RevertAid First Strand cDNA Synthesis Kit (Thermo Fisher Scientific) according to the manufacturer’s instructions. The synthesized cDNA was diluted 1:10 prior to qRT-PCR analysis. qRT-PCR was performed using the ABI® 7500 Real-Time PCR system and the QuantiNova SYBR Green Kit (Qiagen). The relative expression of each gene was normalized to 18S expression. Gene expression was analyzed using the ΔΔCt method. After 7 days of treatment with doxycycline, M2 and 4F cells were harvested and analyzed for the expression of melanocytic and neuronal markers. Moreover, their proliferative and metastatic capacity was examined.

### Western blot

Proteins were extracted from M2 and 4F cells after 7 days of doxycycline treatment, using RIPA buffer (Invitrogen) containing complete mini protease inhibitor cocktail (Roche). The protein concentration was measured using the Pierce BCA Protein Assay kit. The following primary antibody dilutions were used in 5% bovine serum albumin (BSA) in TBST: ASCL1 (a kind gift from Dr. Moritz Mall from the DKFZ, 1:100), BRN2 (Thermo Fisher Scientific Cat# MA5-35032, RRID:AB_2848937, 1:1000), MYT1L (a kind gift from Dr. Moritz Mall from the DKFZ, 1:1000), NEUROD1 (Thermo Fisher Scientific Cat# MA5-32626, RRID:AB_2809903, 1:1000), S100 (Abcam Cat# ab109252, RRID:AB_10862910, 1:1000), MITF (Thermo Fisher Scientific Cat# MA5-32554, RRID:AB_2809831, 1:1000), KI67 (Invitrogen, MA5-14520, RRID AB_10979488, 1:1000), TRP2 (Santa Cruz Biotechnology Cat# sc-74439, RRID:AB_1130818, 1:1000), Melan-A (Agilent Cat# M7196, RRID:AB_2335691, 1:1000) and α-actinin (Santa Cruz Biotechnology Cat# sc-17829, RRID:AB_626633, 1:5000). The secondary antibodies (Cell Signaling Technology Cat# 7076, RRID:AB_330924 and Cell Signaling Technology Cat# 7074, RRID:AB_2099233) were diluted 1:10.000 in 5% BSA in TBST and incubated at RT for 1 h.

### Light and fluorescent microscopy

To evaluate morphological alterations of 4F cells, M2 and 4F cells were seeded into 10 cm culture dishes and treated with doxycycline for more than 7 days. On the fourth day, 4F cells were treated with palbociclib (10 µM) in order to enrich neuron-like cells. On days 4, 7, and 14, pictures were taken with a DM LS Light Microscope (Leica). Fluorescent images were acquired using the Zeiss LSM 700 from the Light Microscopy Core Facility at the DKFZ. The number of fluorescent cells (positive staining for neuron markers) or the number of granules per cell (gH2AX immunostaining detection) were counted using FIJI, with the script developed by the Light Microscopy Core Facility at the DKFZ.

### Immunofluorescence for neuron markers

2 × 10^4^ cells were seeded in 8-chamber culture slides (Falcon). After 7 days of doxycycline treatment, M2 and 4F cells were fixed in 4% paraformaldehyde (PFA) for 5 min on ice and an additional 10 min at RT and then permeabilized with 0.1% Triton X-100 for 10 min. Next, cells were preincubated for 1 h with blocking solution (3% BSA) before administering the primary antibodies anti-b-tubulin III (Sigma-Aldrich Cat# T8578, RRID:AB_1841228) and anti-MAP2 (Sigma-Aldrich Cat# HPA012828, RRID:AB_1853946), respectively, and incubating overnight at 4 °C. The following day, cells were washed and incubated with goat anti-rabbit IgG (H+L) Highly Cross-Adsorbed Secondary Antibody, Alexa Fluor Plus 647 (Thermo Fisher Scientific Cat# A32733, RRID:AB_2633282) and goat anti-mouse IgG (H+L), Superclonal™ Recombinant Secondary Antibody, Alexa Fluor™ 488 (Thermo Fisher Scientific, A28175) for 1 h at RT in the dark. Nuclei were stained with 4, 6-diamidino-2-phenylindole (DAPI, Roche) for 15 min. Slide mounting was performed with fluorescence mounting medium (Dako).

### Cell proliferation assay

Cell viability was analyzed using the BrdU Cell Proliferation ELISA Kit (Abcam, ab126556). 5 × 10^3^ melanoma cells were plated in flat-bottom 96-well plates (Gibco Life Technologies). BrdU was added 8 h before measurement. 24, 48, 72, and 96 h after seeding the cells, they were fixed using Fixing Solution. The fixed plates were stored at 4 °C and tested within 2 weeks. The detector antibody, peroxidase goat anti-mouse IgG Conjugate, TMB Peroxidase Substrate, and Stop Solution were added subsequently according to the manufacturer’s instructions. The plates were tested using an Infinite M1000Pro Microplate Reader with an excitation wavelength of 450 nm and an emission wavelength of 590 nm. Cell Proliferation status was calculated from the resulting change in fluorescence intensity normalized to the value of the original melanoma cell group.

### Colony formation assay

Parental cells, M2 cells treated with doxycycline for 4d (M2-Dox (+)4d), untreated 4F cells (4F-Dox (−)), 4F cells treated with doxycycline for 4d (4F-Dox (+)4d), and 4F cells treated with doxycycline for 4d and palbociclib (10 µM) for 3d (4F-Dox (+)4d-Pal (+)3d) cells were seeded at a density of 50-150 cells per well in a 6-well plate. Doxycycline was added to M2-Dox (+)4d, 4F-Dox (+)4d, and 4F-Dox (+)4d-Pal (+)3d cells, but no palbociclib was added. The old medium was removed and replaced with fresh medium every 3 days. After 5–10 days of induction, the M2-Dox (+), 4F-Dox (+), and 4F-Dox (+)-Pal (+) cells were given normal MEF medium without doxycycline. After 7 to 16 days, cell colonies were stained using crystal violet solution (0.5%). Relative colony area density was determined using ImageJ software (Fiji, RRID:SCR_003070).

### Migration assay

Parental cells, M2-Dox (+)4d, 4F-Dox (−), 4F-Dox (+)4d, and 4F-Dox (+)4d-Pal (+)3d cells were cultured at a density of 2 × 10^4^ to 3 × 10^4^ cells per chamber of an ibidi 2-well culture chamber (Gräfelfing, Germany) for 24 h. After removing the insert, cell migration was monitored every 3 h. Images were acquired 20–24 h after removal of the insert. Acquired images were analyzed using TScratch software.

### Invasion assay

The Cultrex BME 24-well kit (3455–024-K, R&D Systems, Minneapolis, Minnesota) was used for cell invasion assays with A375 and MA1. Cells were starved overnight and then seeded out (3 × 10^4^ cells) in a BME-coated chamber with serum-free medium. Then, the number of invasive cells was measured by calcein staining. Due to the discontinuation of the production of the above kit, the Cell Invasion Assay (Basement Membrane), 96-well, 8 µm (Abcam, ab235697) was used for the SKMel103 and HT144 cell lines according to the manufacturer’s instructions. With both kits, we included parental melanoma cells, M2-Dox (+)4d, 4F-Dox (−), 4F-Dox (+)4d, and 4F-Dox (+)4d-Pal (+)3d cell groups.

### Cell apoptosis assay

Cells were seeded in 6-well plates at a density of ~60%. The following day, M2-Dox (+)4d and 4F-Dox (+)4d cells were treated once with 3 Gy of ionizing radiation. Untreated samples served as negative controls. After 96 h of incubation, adherent cells and cells in the supernatant were collected in tubes, followed by washing with ice-cold PBS. Next, 1 × 10^5^ cells were suspended in 1X Annexin V Binding buffer (100 μL), and 2.5 μL FITC Annexin V and 1 μL of DAPI were added to the cell suspension. Cell solution was gently vortexed and incubated at RT for 20 min, avoiding light exposure. Then, 400 μL of 1X Annexin V Binding buffer was added to each tube, and samples were analyzed by flow cytometry in the Flow Cytometry Core Facility at the DKFZ. FlowJo cell analysis software (RRID:SCR_008520) was used to analyze the data.

### gH2AX immunostaining

1–1.5 × 10^5^ M2 and 4F cells were seeded in 12-well plates on cover slips. After doxycycline treatment for 4d, M2 and 4F cells were subjected once to 3 Gy of ionizing radiation. The untreated sample served as a negative control. After 0.5, 1, 6, and 24 h, cells were fixed in 4% paraformaldehyde (PFA) for 15 min at RT and then permeabilized with 0.2% Triton X-100 for 8 min. Next, cells were preincubated for 1 h with blocking solution (5% BSA in 0.05% Triton PBS) before incubation with the primary antibody anti-phospho-Histone H2AX (Ser139) (Millipore Cat# 05-636, RRID:AB_309864) overnight at 4 °C. The following day, cells were washed and incubated with goat anti-mouse IgG (H+L), Superclonal™ Recombinant Secondary Antibody, Alexa Fluor™ 488 (Thermo Fisher Scientific, A28175) for 1 h at RT in the dark. Nuclei were stained with 4, 6-diamidino-2-phenylindole (DAPI, Roche) for 15 min. Slide mounting was performed with fluorescence mounting medium (Dako).

### Mouse experiments

Tumorigenicity and metastatic potential of transdifferentiated HT144-4F and A375-4F were determined in vivo by injecting 2–5 × 10^6^ cells subcutaneously into the flanks of NOD scid gamma (NSG) mice. The expression of the transdifferentiation factors was maintained by adding doxycycline to the drinking water (2 mg/ml, Sigma-Aldrich, Cat. No. D9891). The tumor size was measured every day. When the size reached 1.5 cm in diameter, mice were euthanized. For the in vivo metastasis assay, 1.5–5 × 10^6^ cells were injected into the tail veins of NSG mice. 6 weeks later, the mice were euthanized, and lungs and livers were collected. Lung and liver samples were fixed, and the metastases were counted. Both male as well as female animals with an age of at least 12 weeks (adult) were used for the experiments, and they were assigned to different experimental groups through randomization. The number of animals necessary to achieve statistically significant conclusions was determined with the help of a simulation conducted with the software R, version 4.2.2, after consultation of the Biostatistics core facility of the German Cancer Research Center. No blinding was employed in the animal experiments.

### Immunohistochemistry

Hematoxylin-eosin (HE) staining was done for each paraffin sample of subcutaneous tumors, livers, or lungs. For immunohistochemistry, 4 μm-thick sections of the sample were stained with target antibodies S100 (Agilent Cat# Z0311, RRID:AB_10013383), KI67 (Abcam Cat# ab16667, RRID:AB_302459), Melan-A (Novus Cat# NBP1-30151, RRID:AB_1987285) according to the manufacturers’ instructions. Simultaneously, a negative control without the first antibody and a verified positive control were stained to avoid false-positive or -negative results.

### RNA sequence profiling

Total RNA from three independent experiments was extracted using the RNeasy mini kit (QIAGEN) according to the manufacturer’s protocol. A NanoDrop ND-1000 spectrophotometer was used to determine the quality and quantity of the RNA. The extracted RNAs were sent to BGI Company (Hong Kong) for sequencing. They constructed the Strand Specific RNA library (Eukaryote, oligo dT, mRNA enrichment), sequenced using the DNBSEQ platform (Paired End 100 bp sequencing, 20 M clean read pairs (4 G data) per sample), and filtered the raw data with adapter sequences or low-quality sequences. This filter step was completed by SOAPnuke software (RRID:SCR_015025) [13] in the Galaxy platform [14], reads from each sample replicate were mapped to the hg19 human genome using HISAT2. Then, uniquely mapped reads were processed with featureCounts and DESeq2 (RRID:SCR_000154) for differential gene expression analysis [15–17]. Ingenuity Pathway Analysis (IPA) software was used for the generation of graphs depicting differentially expressed genes.

### DNA methylation assay

DNA samples were extracted from M2-Dox (+)7d and 4F-Dox (+)7 d cell lines with QIAGEN DNeasy Blood & Tissue Kit, and sample quality was assessed by NanoDrop ND-1000 spectrophotometer. The microarray unit of the DKFZ Genomics core facility conducted gene expression profiling using Illumina Infinium MethylationEPIC BeadChip. DNA methylation processing, including QC, probe filtering, normalization, dimensionality reduction analysis, and differentially methylated probes analysis, has been performed using ChaMP package (version 2.18.3) in the R environment (version 4.0.1). CNV analysis was performed using minfi (v1.34.0) and conumee (v1.9.0-1) packages.

### Statistical analysis

Experiments were conducted at least in biological triplicate. Data were presented as box plots displaying individual data points, with whiskers extending from minimum to maximum values. Statistical comparisons were performed using an unpaired *t*-test, with significance set at *P* < 0.05. Except for sequencing data, all statistical analyses were performed with GraphPad Software (RRID:SCR_002798).

## Results

### Melanoma cells can be transdifferentiated into neuron-like cells

In order to examine if melanoma cells can be transdifferentiated into neurons, the neuron-specific transcription factors ASCL1, BRN2, MYT1L, and NEUROD1 were ectopically overexpressed in different melanoma cell lines (Figs. 1B, C and S1A, B). For this purpose, melanoma cells were transduced with doxycycline-inducible, lentiviral vectors harboring the transgenes, and expression was induced by applying doxycycline. Already after a few days, some remarkable changes in morphology could be observed. Parental HT144 melanoma cells grew in tightly packed colonies. Single cells had a rather compact or slightly spindle-shaped morphology. However, already after only 7 days of doxycycline treatment, many cells with rather small, round or triangular somata and long processes could be found. In order to enrich for neuron-like cells, we additionally treated the cells with ROCK inhibitor (10 µM) and palbociclib (10 µM) [18] for 10 days. Palbociclib inhibited the rapid proliferation of melanoma cells, leading to cell cycle arrest and eventual elimination. In contrast, neuron-like cells, which have already differentiated and exhibited low proliferation, remained unaffected, resulting in their selective enrichment. ROCK inhibitor, on the other hand, was specifically utilized to facilitate the morphological development of neuron-like cells in those experiments aimed at visualizing morphological changes (Figs. 1D and S2). In long-term cultures, it helped neuron-like cells to develop a more pronounced neuronal morphology. Without ROCK inhibitor, neurons are less likely to extend synapses and exhibit neuronal characteristics. Interestingly, the neuron-like cells reached a stable state after 14 days of doxycycline treatment combined with 10 days of palbociclib selection. This means that the cells kept the neuron-like phenotype even upon doxycycline withdrawal, and they did not proliferate anymore (Fig. 1D). With this approach, we successfully transdifferentiated *BRAF*-mutated (A375, HT144, MA1) and *NRAS*-mutated human melanoma cells (SKMel30, SKMel103) as well as murine Ret melanoma cells derived from *Ret* transgenic mice [19] (Fig. S2). In order to calculate the efficiency of the transdifferentiation process, we performed stainings against the neuronal markers b-III tubulin (TUJ1) and MAP2 and quantified the number of positive cells [20]. This quantification revealed an efficiency of 2–5% (Figs. 1F and S3A, B) [21]. On the molecular level, the expression of the neuronal markers hMYT1L, hBRN2, TUJ1, MAP2, SYT5, and NeuN increased significantly in different cell lines, as could be shown by qPCR (Figs. 1E and S3C). Immunofluorescence staining revealed that transdifferentiated cells were positive for MAP2 and/or TUJ1, with MAP2 mostly expressed in the cell somata, and TUJ1 mostly expressed in the processes (Fig. 1F).

### Transdifferentiation alters the global gene expression of melanoma cells

**Fig. 2:**
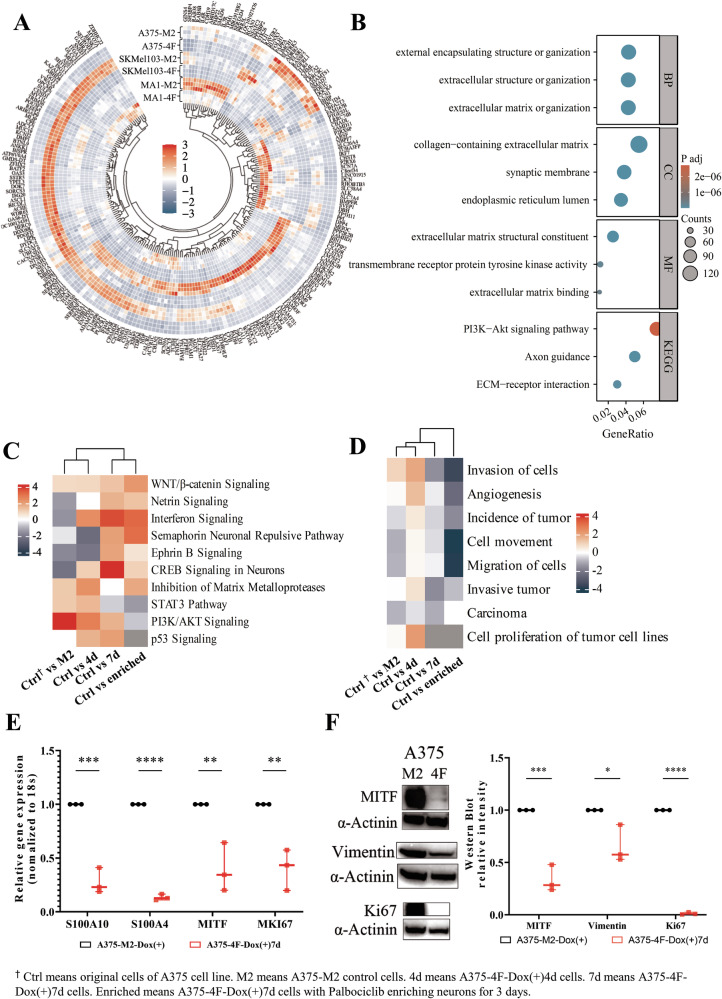
RNAseq analysis of cells during and after transdifferentiation. **A** Heatmap of 215 differentially expressed genes (DEGs) with the same trend in 3 cell lines. **B** GO-KEGG analysis of DEGs between the A375 and A375-4F-Dox (+)7d-Pal (+)3d group. **C** Heatmap of canonical pathways enriched. From left to right: A375 vs A375-M2, A375 vs A375-4F-Dox (+)4d, A375 vs A375-4F-Dox (+)7d, A375 vs A375-Dox (+)7d-Pal (+)3d. **D** Heatmap of disease and function analysis. From left to right: A375 vs A375-M2, A375 vs A375-4F-Dox (+)4d, A375 vs A375-4F-Dox (+)7d, A375 vs A375-Dox (+)7d-Pal (+)3d. **E** qPCR analysis showed a downregulation of melanoma markers 7 days upon induction of transdifferentiation with doxycycline. **F** Melanoma markers were also downregulated at the protein level upon treatment with doxycycline for 7 days.

In order to examine alterations in global gene expression during transdifferentiation, we performed RNA sequencing on samples from different melanoma cell lines (A375, SkMel103, and MA1) that were treated with doxycycline for 4 (4F-Dox (+)4d) and 7 days (4F-Dox (+)7d), respectively. Cells that were not transduced with the transdifferentiation factors but still treated with doxycycline (M2-Dox (+)) served as a control. PCA analysis demonstrated that although the cell lines used for the experiments differed from each other in terms of global gene expression, the transdifferentiation process initiated a comparable shift in global gene expression in each cell line (Fig. S4A). When comparing the M2-Dox (+)7d and 4F-Dox (+)7d groups, we observed that the pattern of differentially expressed genes varied from cell line to cell line (Fig. S4C). However, we found that 331 genes were differentially expressed in all three cell lines (Fig. S4B). Among these 331 genes, 215 genes showed the same trend of up- or downregulation in the 4F group compared to the M2 group (Fig. 2A). Gene Ontology and Kyoto Encyclopedia of Genes and Genomes (GO-KEGG) analysis of these 215 genes demonstrated that they were mostly related to neuronal structures and functions (Fig. S4E), e.g., regulation of membrane potential, integral components of the synaptic membrane, the presynaptic active zone, adrenergic signaling, etc. GO-KEGG analysis based on the most significant DEGs in the 4F-Dox (+)7d-Pal (+)3d group compared to the parental cells confirmed that these genes are connected to neuronal pathways and structures including axon guidance, synaptic membrane, axon genesis (Fig. 2B). IPA showed a gradual upregulation of genes involved in neuron-related signaling pathways from 4F-Dox (+)4d to 4F-Dox (+)7d. Neuron-like cells enriched by palbociclib treatment showed a similar activation of neuron-related pathways as 4F-Dox (+)7d cells. On the other hand, tumor-growth-related pathways like the STAT3 and PI3K/AKT pathways were less active in the 4F-Dox (+)7d group and even more significantly downregulated in 4F-Dox (+)7d-Pal (+)3d cells (Fig. 2C). DNA methylation profiling of the M2-Dox (+)7d and 4F-Dox (+) 7d group of A375, SKMel103 and MA1 cells revealed a similar clustering as transcriptome analysis (Fig. S5A, B). Upon transdifferentiation, A375 had the most prominent DNA methylation alterations while MA1 exhibited the least alterations (Fig. S6A). The heatmap for the top variable and significant (*p* < 0.05) CpGs showed overlapping methylation changes at some sites across all 3 cell lines (Fig. S6B), with the 4F cells clustering together based on the top 40 significant CpGs (Fig. S5C). Among the most significantly altered CpGs in genes in A375 (Fig. S6E), GO-KEGG analysis showed the enriched functions, pathways, and structures to be related to neurons (Fig. S5D). Differentially methylated probe analysis of SKMel103 and MA1 cells presented only a few differentially methylated genes, and, for these cases, the GO-KEGG analysis did not report any enriched terms. (Fig. S6C, D). Having shown that melanoma cells could be transdifferentiated into neuron-like cells, we wanted to know whether this process also changed some tumor-related cell characteristics. The RNAseq analysis showed that the expression of a variety of neuronal markers was upregulated upon transdifferentiation, while melanoma or tumor-related markers were significantly downregulated (Fig. S4D, F). This finding was further confirmed by qPCR. In transdifferentiated A375 cells the melanoma markers MKI67, MITF, S100A4, S100A10, MMP2 and MMP9 were significantly downregulated (Figs. 2E and S7A). Transdifferentiated MA1 showed a significantly reduced expression of S100A11, S100A10, S100A4, and MITF, while transdifferentiated SKMel103 expressed significantly less MMP9, S100A4, S100A11, and MKI67. In transdifferentiated HT144, the expression of MITF, S100A4, and S100A10 was significantly downregulated (Fig. S7A). The most frequently downregulated genes among the 4 cell lines were the melanoma markers MITF, S100, and KI67, which are useful for diagnostic or prognostic purposes [22, 23]. Western blot analyses confirmed the downregulation of these melanoma markers on the protein level (Fig. S7B–D). Furthermore, the disease and functional analysis based on the RNAseq data results also demonstrated that the expression of genes related to tumor incidence, invasion of cells, invasive tumor, cell movement, migration of cells, angiogenesis, and proliferation of tumor cells were reduced already after 4 days of doxycycline treatment, even more reduced after 7 days of doxycycline treatment, and drastically reduced after 7 days of doxycycline treatment and selection with palbociclib (Fig. 2D).

### Transdifferentiation reduces the tumorigenicity of melanoma cells in vitro

**Fig. 3:**
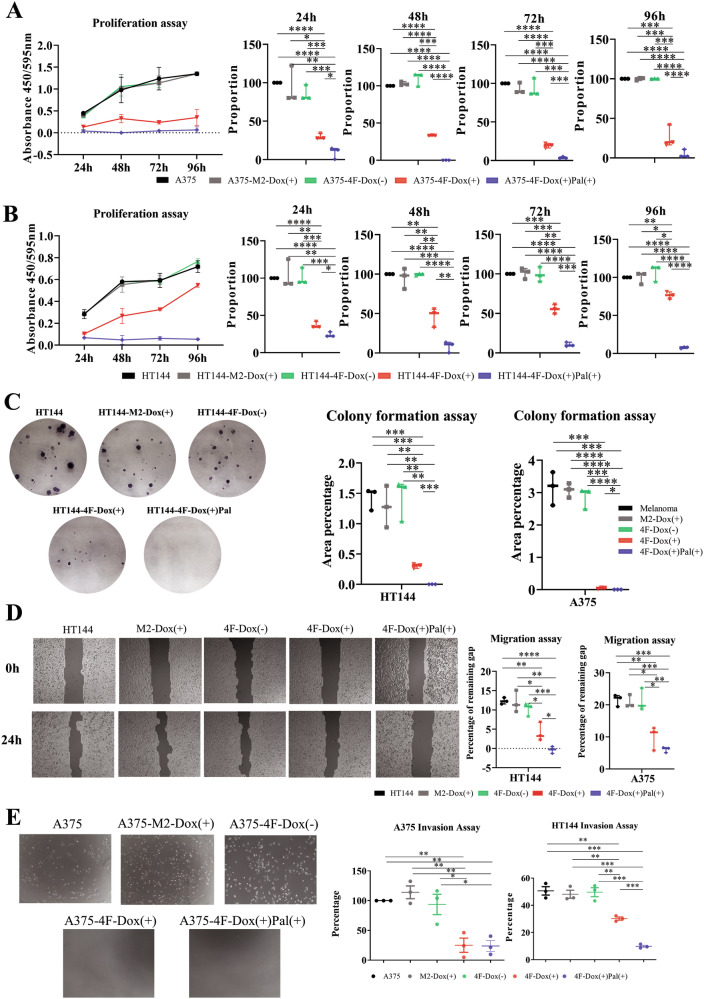
Loss of tumor cell properties in vitro. **A**, **B** Transdifferentiated melanoma cells exhibited reduced proliferation capacity compared to their M2-vector counterparts. Transdifferentiated cells after enrichment exhibited almost no proliferation capacity. **C** Colony formation assay revealed that transdifferentiated-4F cells were much less tumorigenic, while transdifferentiated and enriched 4F cells showed no tumorigenicity. A375-M2 and -4F were treated with doxycycline for 5 days followed by 2 days without doxycycline. HT144-M2 and 4F were treated with doxycycline for 10 days, followed by 6 days without doxycycline. **D** Migration capacity was markedly reduced in the 4F-Dox (+) group and even more reduced in the palbociclib-treated, enriched group. **E** Invasion capacity was significantly reduced in the 4F-Dox (+) as well as the 4F-Dox (+)-Pal (+) group.

After demonstrating that transdifferentiation induces profound changes on the molecular level, we performed functional studies to verify whether these changes also go along with an altered tumorigenicity of the melanoma cells. Using a BrdU proliferation assay, we analyzed the proliferative capacity of the neuron-like cells in comparison to the parental melanoma cells, showing that the neuron-like cells exhibited a drastically reduced proliferative capacity. Moreover, we could see a significant difference between those neuron-like cells that were selected and enriched with palbociclib versus those that were not treated with palbociclib after 4 days of doxycycline treatment (Figs. 3A, B and S8A, B). Next, we assessed the tumorigenicity of the neuron-like cells with a colony formation assay. While the parental melanoma cells formed numerous big colonies, the non-enriched neuron-like cells formed much smaller and less colonies after doxycycline withdrawal. However, enriched neuron-like cells did not form any colonies even after doxycycline withdrawal. The difference in the percentage of area covered compared between every two groups was significant (Figs. 3C and S8C). Additionally, we utilized a migration assay to assess the migrative capacity of neuron-like cells. Our results indicate that both enriched and non-enriched cells have significantly less migrative capacity compared to the parental melanoma cells, and the enriched cells exhibit the least migration (Figs. 3D and S8D). Finally, we quantified the invasive capacity with a cell invasion assay to see if neuron-like cells are able to degrade extracellular matrix proteins and invade an artificial basement membrane. Our results clearly demonstrated that both enriched and non-enriched cells exhibited almost no invasive capacity compared to the parental melanoma cells (Figs. 3E and S8E). These results show that transdifferentiated, neuron-like cells were significantly less tumorigenic, less migrative, and less invasive. Moreover, these features of tumor cell features were nearly completely abrogated in the palbociclib-treated, enriched neuron-like cells.

### Transdifferentiation reduces the tumorigenicity and metastatic capacity of melanoma cells in vivo

**Fig. 4:**
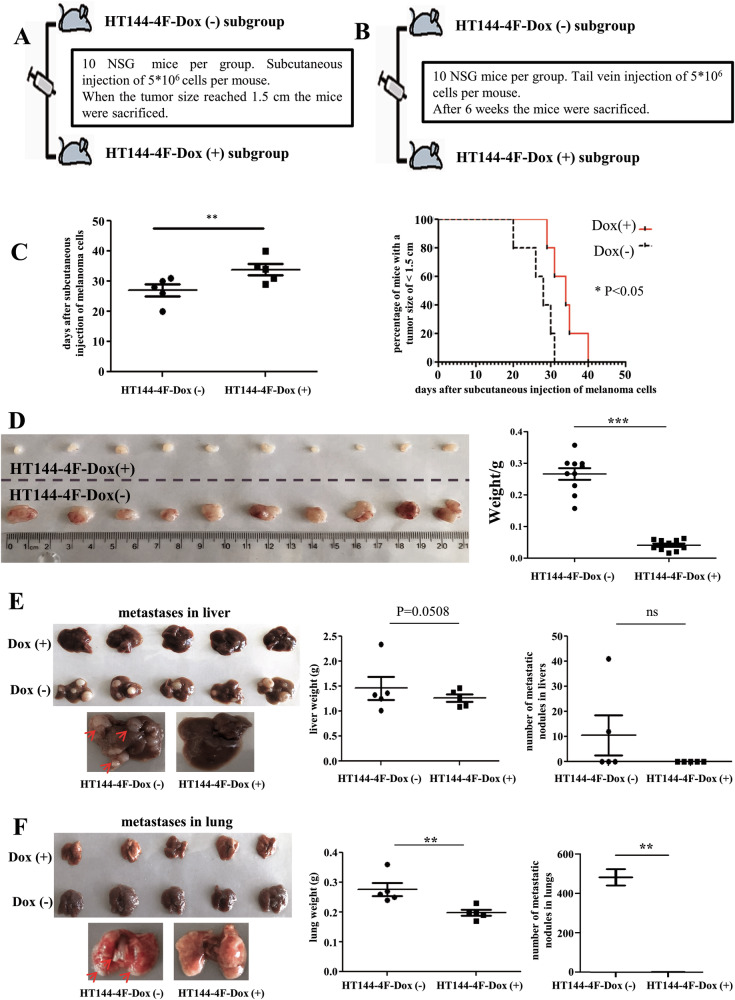
Reduced tumorigenic and metastastatic capacity of transdifferentiated HT144 melanoma cells in vivo. **A**, **B** Schematic overview of the in vivo experimental procedure. **C** Mice injected with transdifferentiated cells exhibit a longer survival time than those injected with the parental melanoma cells. **D** Comparison of size and weight of tumors derived from transdifferentiated or parental melanoma cells upon subcutaneous injection into NSG mice. **E** Comparison of the number of visible metastases and liver weight upon intravenous injection of transdifferentiated parental melanoma cells into NSG mice. **F** Comparison of the number of visible metastases and lung weight upon intravenous injection of transdifferentiated or parental melanoma cells into NSG mice.

Since we could show that the tumorigenicity of transdifferentiated, neuron-like cells was significantly reduced in vitro, we decided to investigate the tumorigenicity and metastatic capacity of those cells in vivo (Figs. 4A, B and S9A). To evaluate the tumor growth, parental and transdifferentiated melanoma cells were subcutaneously injected into immunocompromised mice. Remarkably, those mice injected with transdifferentiated cells only developed very small tumors (Figs. 4D and S9B), while mice injected with parental melanoma cells developed big tumors and had to be euthanized within a month (Fig. 4C). HE staining of the excised tumors showed that the tumors derived from parental melanoma cells started to infiltrate the surrounding fat tissue and that the tumor cells had bigger, hyperchromatic nuclei and more fissions. In contrast, tumors derived from transdifferentiated cells were more superficial and had a lower tumor cell density (Fig. S10A). In addition to tumor growth, the metastasizing potential of the transdifferentiated versus parental cells was assessed. Cells were injected into the tail veins of immunocompromised mice. Six weeks later, the mice were euthanized, and lungs and livers were excised, fixed, and visible metastases were counted. While the injection of parental melanoma cells resulted in nicely visible metastases in the liver, the transdifferentiated cells did not cause any liver metastases (Figs. 4E and S9C). Accordingly, the average liver weight was lower in mice injected with transdifferentiated cells (Fig. 4E). Likewise, the average lung weight was much higher after injection of parental melanoma cells compared to the injection of transdifferentiated cells indicating the presence of metastases in the lungs of mice injected with the parental melanoma cells (Figs. 4F and S9C). The number of metastases in the liver as well as the number of metastases in the lung was much lower upon injection of transdifferentiated cells (Fig. 4F). HE staining also revealed almost no metastases in the livers or lungs of mice injected with transdifferentiated cells (Fig. S10C, D). Taken together, our in vitro and in vivo studies demonstrated that the melanoma cells were significantly less tumorigenic after transdifferentiation.

### Transdifferentiation increases the sensitivity to radiotherapy

**Fig. 5:**
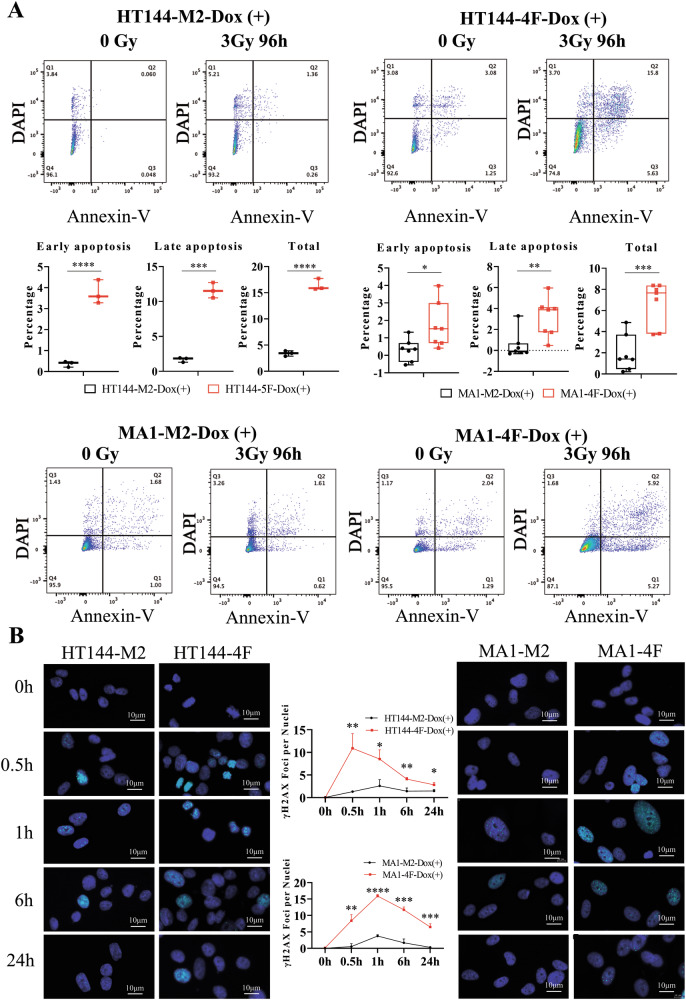
Increased sensitivity of transdifferentiated cells to radiotherapy. **A** Quantification of the percentage of apoptotic HT144 and MA1 melanoma cells 96 h after irradiation with 3 Gy. Early apoptotic cells (Annexin V⁺/DAPI^−^) appear in the lower right quadrant, indicating phosphatidylserine externalization with an intact membrane. Late apoptotic cells (Annexin V⁺/DAPI⁺) are in the upper right quadrant, characterized by both phosphatidylserine exposure and membrane permeability. Necroptotic cells (Annexin V^−^/DAPI⁺) are in the upper left quadrant, showing membrane rupture without phosphatidylserine externalization. The percentage of apoptotic and dead cells was much higher among the transdifferentiated cells in comparison to the parental melanoma cells. **B** gH2AX analysis 0.5, 1, 6, or 24 h after single treatment of HT144 and MA1 melanoma cells with 3 Gy radiation.

Targeted therapy and immunotherapy are usually recommended as the first-line therapeutic treatment for advanced melanoma, while radiotherapy is usually used as an adjuvant therapy [24, 25]. We could show that the transdifferentiation process altered melanoma cell characteristics connected to tumorigenicity. Thus, we wanted to investigate if transdifferentiation affects the susceptibility of melanoma cells toward radiation therapy. We utilized the Annexin V-DAPI apoptosis assay to examine how transdifferentiated, neuron-like cells react to combinational radiation treatment compared to the parental melanoma cells. In general, 96 h after being subjected to 3 Gy radiation, the percentage of apoptotic cells was much higher among transdifferentiated cells compared to the parental melanoma cells. This was observed for all 4 melanoma cell lines that were tested (Figs. 5A and S11A–C). Quantifying the DNA damage after radiation with the help of gH2AX immunofluorescence staining [26] demonstrated that transdifferentiated cells exhibited much more damage than the parental cells after 0.5 or 1 h of irradiation with 3 Gy, and recovered much worse after 6 and 24 h (Figs. 5B, C and S11D, E). These assays revealed that transdifferentiation significantly increased the sensitivity of the melanoma cells toward radiation treatment.

## Discussion

In our study, we have shown that human melanoma cells can successfully be transdifferentiated to neuron-like cells that express neuronal instead of melanocytic markers and that show a morphology resembling that of neuronal cells. Moreover, we were able to demonstrate that the proliferation, colony formation, migration, and invasion capacities of the melanoma cells were drastically decreased in vitro upon transdifferentiation. In line with this, in vivo experiments revealed that the tumorigenicity after transdifferentiation was reduced markedly, as indicated by significantly less tumor growth and metastasizing potential. Interestingly, transdifferentiated melanoma cells were also more sensitive to radiotherapy. Our results are in good agreement with previously published studies showing that transdifferentiation of cancer cells is possible and that this affects the tumorigenicity of the cells [3, 4]. Ectopic overexpression of the four factors ASCL1, BRN2, MYT1L, and NEUROD1 resulted in a change in the transcriptional network toward a neuron-specific expression pattern. As reported by Wapinski and colleagues, ASCL1 acts as a pioneer factor that binds to its physiologic neural targets in closed chromatin and initiates the transformation toward a neuronal phenotype. ASCL1 also recruits BRN2 to specific binding sites and thereby enables neuron-specific gene expression [27]. In contrast, MYT1L directly suppresses various somatic lineage expression programs, but not the neuronal program, and in this way promotes neuronal differentiation [28]. On the other hand, MYT1L possesses the capability to suppress proliferative pathways, such as WNT and NOTCH, in human cells [29]. We assume that this mechanism also allowed the melanoma cells used in this study to change toward neuron-like cells in a similar fashion. Together, the four factors suppressed melanocytic gene expression and set up neuron-specific gene expression, including the expression of their endogenous counterparts. Apart from altering the global gene expression pattern and the morphology of the melanoma cells, transdifferentiation also had a measurable impact on their functional properties. Proliferation and migration were reduced dramatically, as one would expect from terminally differentiated neuronal cells. The mechanisms that promote the growth and migration of melanoma cells can only be effective within the context of a melanocyte/melanoma-specific expression network. Switching to a neuron-specific transcriptional network unhinges certain growth-promoting pathways, nullifying their effects. For this reason, the colony formation capacity of melanoma cells was completely abrogated upon transdifferentiation. Accordingly, the tumorigenic and metastatic capacities of the transdifferentiated melanoma cells were also greatly reduced in vivo compared to the parental melanoma cells, confirming that the melanoma-specific tumorigenicity cannot come into effect in a neuron-specific transcriptional background. However, both the tumors derived from parental as well as transdifferentiated cells showed strong melanocytic properties. This suggests that the population of 4F-Dox (+) cells without palbociclib-enrichment contains melanoma-like cells, which are responsible for the tumor formation in vivo. HE staining demonstrated that tumors derived from 4F-Dox (+) cells showed a comparably high amount of cells positive for the proliferation marker KI67, like tumors derived from parental melanoma cells (Fig. S10B). This observation is in accordance with our in vitro proliferation study results showing that 4F-Dox (+) cells that have not been selected with palbociclib were still tumorigenic and proliferated in general, albeit at partly significantly lower rates compared to the control groups that were not subjected to Dox treatment (Figs. 3A–C and S8A–C). We hypothesize that the transdifferentiation efficiency was less than 100% and yielded a heterogeneous cell population consisting of induced neurons, which were unable to proliferate and form tumors, induced neuron-like cells, which retained proliferative capacity but lost melanoma features, and cells that were stuck in between a melanoma and induced neuron-like phenotype, which retained some melanoma markers, were more proliferative compared to induced neuron-like cells, and which formed tumors and metastasized in vivo. Having shown that transdifferentiation drastically mitigated the tumorigenicity of melanoma cells, we also investigated if the transdifferentiated cells were more susceptible to radiation therapy compared to the parental cells, which are usually resistant to radiation. Indeed, we were able to prove that the transdifferentiating cells showed more sensitivity to radiotherapy compared to the parental melanoma cells. However, the exact mechanism behind this resensitization remains to be studied. Moreover, it would be interesting to investigate if the transdifferentiated cells react differently to targeted therapy and immunotherapy than the parental melanoma cells. Based on the results presented in this study, transdifferentiation of melanoma cells could have the potential to complement the arsenal of already established therapeutic options for the treatment of melanoma. On the one hand, the abrogation of the tumorigenic properties of melanoma cells upon transdifferentiation would be very beneficial for obvious reasons. On the other hand, restoring the susceptibility of melanoma cells toward radiation therapy or potentially targeted and immune therapy would increase the efficacy of established therapeutic measures enormously. In the case of locally restricted tumors, replication-deficient viral particles transmitting the necessary factors for transdifferentiation could be topically applied by injection, comparable to other intralesional therapeutic approaches like the use of the oncolytic T-VEC [30]. The use of viral particles with a tropism that is restricted to melanoma cells could even enable a systemic delivery of the transdifferentiation factors to melanoma metastases throughout the body [31]. In summary, we could show in this study that melanoma cells can be transdifferentiated into neuron-like cells that express neuronal markers and adopt a neuron-like morphology. During this process, these cells lose their tumorigenic potential and become sensitive to radiotherapy. We demonstrated that the tumorigenic phenotype and sensitivity to radiotherapy were associated with the lineage of origin, and we suggest that altering the lineage of origin of melanoma cells by means using different approaches, such as gene therapy or pharmacological compounds, could represent a new therapeutic alternative for the treatment of malignant melanoma.

## Supplementary information

Supplemental Material

western blots

qPCR results

## Data Availability

The data that support the findings of this study are available from the corresponding author upon request. Gene expression data sets were uploaded to the Gene Expression Omnibus (GEO) (RRID:SCR_005012) database (GSE243890). https://www.ncbi.nlm.nih.gov/geo/query/acc.cgi?acc=GSE243890. DNA methylation data sets were uploaded to the GEO database (GSE244106). https://www.ncbi.nlm.nih.gov/geo/query/acc.cgi?acc=GSE244106.

## References

[1] Mishra H, Mishra PK, Ekielski A, Jaggi M, Iqbal Z, Talegaonkar S. Melanoma treatment: from conventional to nanotechnology. J Cancer Res Clin Oncol. 2018;144:2283–302. 30094536 10.1007/s00432-018-2726-1 PMC11813321

[2] Grath A, Dai G. Direct cell reprogramming for tissue engineering and regenerative medicine. J Biol Eng. 2019;13:14. 30805026 10.1186/s13036-019-0144-9 PMC6373087

[3] Rapino F, Robles EF, Richter-Larrea JA, Kallin EM, Martinez-Climent JA, Graf T. C/EBPα induces highly efficient macrophage transdifferentiation of B lymphoma and leukemia cell lines and impairs their tumorigenicity. Cell Rep. 2013;3:1153–63. 23545498 10.1016/j.celrep.2013.03.003

[4] Fehrenbach S, Novak D, Bernhardt M, Larribere L, Boukamp P, Umansky V, et al. Loss of tumorigenic potential upon transdifferentiation from keratinocytic into melanocytic lineage. Sci Rep. 2016;6:28891. 27387763 10.1038/srep28891 PMC4937495

[5] Bernhardt M, Novak D, Assenov Y, Orouji E, Knappe N, Weina K, et al. Melanoma-derived iPCCs show differential tumorigenicity and therapy response. Stem Cell Rep. 2017;8:1379–91. 10.1016/j.stemcr.2017.03.007 PMC5425615 28392221

[6] Utikal J, Maherali N, Kulalert W, Hochedlinger K. Sox2 is dispensable for the reprogramming of melanocytes and melanoma cells into induced pluripotent stem cells. J Cell Sci. 2009;122:3502–10. 19723802 10.1242/jcs.054783 PMC2746132

[7] Stricker SH, Feber A, Engström PG, Carén H, Kurian KM, Takashima Y, et al. Widespread resetting of DNA methylation in glioblastoma-initiating cells suppresses malignant cellular behavior in a lineage-dependent manner. Genes Dev. 2013;27:654–69. 23512659 10.1101/gad.212662.112 PMC3613612

[8] Shin D, Cho K-H. Critical transition and reversion of tumorigenesis. Exp Mol Med. 2023;55:692–705. 37009794 10.1038/s12276-023-00969-3 PMC10167317

[9] Vierbuchen T, Ostermeier A, Pang ZP, Kokubu Y, Südhof TC, Wernig M. Direct conversion of fibroblasts to functional neurons by defined factors. Nature. 2010;463:1035–41. 20107439 10.1038/nature08797 PMC2829121

[10] Pang ZP, Yang N, Vierbuchen T, Ostermeier A, Fuentes DR, Yang TQ, et al. Induction of human neuronal cells by defined transcription factors. Nature. 2011;476:220–3. 21617644 10.1038/nature10202 PMC3159048

[11] Yang N, Chanda S, Marro S, Ng Y-H, Janas JA, Haag D, et al. Generation of pure GABAergic neurons by transcription factor programming. Nat Methods. 2017;14:621–8. 28504679 10.1038/nmeth.4291 PMC5567689

[12] Bernhardt M, Orouji E, Larribere L, Gebhardt C, Utikal J. Efficacy of vemurafenib in a trametinib-resistant stage IV melanoma patient-letter. Clin Cancer Res. 2014;20:2498–9. 24789037 10.1158/1078-0432.CCR-13-2349

[13] Chen Y, Chen Y, Shi C, Huang Z, Zhang Y, Li S, et al. SOAPnuke: a MapReduce acceleration-supported software for integrated quality control and preprocessing of high-throughput sequencing data. Gigascience. 2018;7:1–6. 29220494 10.1093/gigascience/gix120 PMC5788068

[14] Galaxy Community. The Galaxy platform for accessible, reproducible and collaborative biomedical analyses: 2022 update. Nucleic Acids Res. 2022;50:W345–51. 35446428 10.1093/nar/gkac247 PMC9252830

[15] Kim D, Langmead B, Salzberg SL. HISAT: a fast spliced aligner with low memory requirements. Nat Methods. 2015;12:357–60. 25751142 10.1038/nmeth.3317 PMC4655817

[16] Liao Y, Smyth GK, Shi W. featureCounts: an efficient general purpose program for assigning sequence reads to genomic features. Bioinformatics. 2014;30:923–30. 24227677 10.1093/bioinformatics/btt656

[17] Love MI, Huber W, Anders S. Moderated estimation of fold change and dispersion for RNA-seq data with DESeq2. Genome Biol. 2014;15:550. 25516281 10.1186/s13059-014-0550-8 PMC4302049

[18] Telezhkin V, Schnell C, Yarova P, Yung S, Cope E, Hughes A, et al. Forced cell cycle exit and modulation of GABAA, CREB, and GSK3β signaling promote functional maturation of induced pluripotent stem cell-derived neurons. Am J Physiol Cell Physiol. 2016;310:C520–41. 26718628 10.1152/ajpcell.00166.2015

[19] Umansky V, Sevko A. Ret transgenic mouse model of spontaneous skin melanoma: focus on regulatory T cells. Pigment Cell Melanoma Res. 2013;26:457–63. 23560814 10.1111/pcmr.12104

[20] Chuang W, Sharma A, Shukla P, Li G, Mall M, Rajarajan K, et al. Partial reprogramming of pluripotent stem cell-derived cardiomyocytes into neurons. Sci Rep. 2017;7:44840. 28327614 10.1038/srep44840 PMC5361100

[21] Ang CE, Wernig M. Induced neuronal reprogramming: Induced neuronal reprogramming. J Comp Neurol. 2014;522:2877–86. 24771471 10.1002/cne.23620 PMC4099045

[22] Jurmeister P, Bockmayr M, Treese C, Stein U, Lenze D, Jöhrens K, et al. Immunohistochemical analysis of Bcl-2, nuclear S100A4, MITF and Ki67 for risk stratification of early-stage melanoma - A combined IHC score for melanoma risk stratification. J Dtsch Dermatol Ges. 2019;17:800–8. 31437373 10.1111/ddg.13917

[23] Ohsie SJ, Sarantopoulos GP, Cochran AJ, Binder SW. Immunohistochemical characteristics of melanoma. J Cutan Pathol. 2008;35:433–44. 18399807 10.1111/j.1600-0560.2007.00891.x

[24] Coit DG, Thompson JA, Albertini MR, Barker C, Carson WE, Contreras C, et al. Cutaneous melanoma, version 2.2019, NCCN Clinical Practice Guidelines in Oncology. J Natl Compr Cancer Netw. 2019;17:367–402. 10.6004/jnccn.2019.0018 30959471

[25] Keilholz U, Ascierto PA, Dummer R, Robert C, Lorigan P, van Akkooi A, et al. ESMO consensus conference recommendations on the management of metastatic melanoma: under the auspices of the ESMO Guidelines Committee. Ann Oncol. 2020;31:1435–48. 32763453 10.1016/j.annonc.2020.07.004

[26] Sharma A, Singh K, Almasan A. Histone H2AX phosphorylation: a marker for DNA damage. Methods Mol Biol. 2012;920:613–26. 22941631 10.1007/978-1-61779-998-3_40

[27] Wapinski OL, Vierbuchen T, Qu K, Lee QY, Chanda S, Fuentes DR, et al. Hierarchical mechanisms for direct reprogramming of fibroblasts to neurons. Cell. 2013;155:621–35. 24243019 10.1016/j.cell.2013.09.028 PMC3871197

[28] Mall M, Kareta MS, Chanda S, Ahlenius H, Perotti N, Zhou B, et al. Myt1l safeguards neuronal identity by actively repressing many non-neuronal fates. Nature. 2017;544:245–9. 28379941 10.1038/nature21722 PMC11348803

[29] Weigel B, Tegethoff JF, Grieder SD, Lim B, Nagarajan B, Liu Y-C, et al. MYT1L haploinsufficiency in human neurons and mice causes autism-associated phenotypes that can be reversed by genetic and pharmacologic intervention. Mol Psychiatry. 2023;28:2122–35. 36782060 10.1038/s41380-023-01959-7 PMC10575775

[30] Kalsi S, Galenkamp AL, Singh R, Khosla AA, McGranaghan P, Cintolo-Gonzalez J. Talimogene laherparepvec (T-VEC) and emerging intralesional immunotherapies for metastatic melanoma: a review. Curr Oncol Rep. 2024;26:1651–63. 39602056 10.1007/s11912-024-01611-9 PMC11646270

[31] Martin F, Neil S, Kupsch J, Maurice M, Cosset F, Collins M. Retrovirus targeting by tropism restriction to melanoma cells. J Virol. 1999;73:6923–9. 10400790 10.1128/jvi.73.8.6923-6929.1999 PMC112777

