# Supplementary material for: Direct transdifferentiation of tumorigenic melanoma cells induces tumor cell reversion
Source: Cell Death Dis. 2025 Jul 25;16(1):563. doi: 10.1038/s41419-025-07863-y (PMC12297470; doi:10.1038/s41419-025-07863-y)
Supplement: Supplementary file 1 — Supplemental Material [file 41419_2025_7863_MOESM1_ESM.pdf]

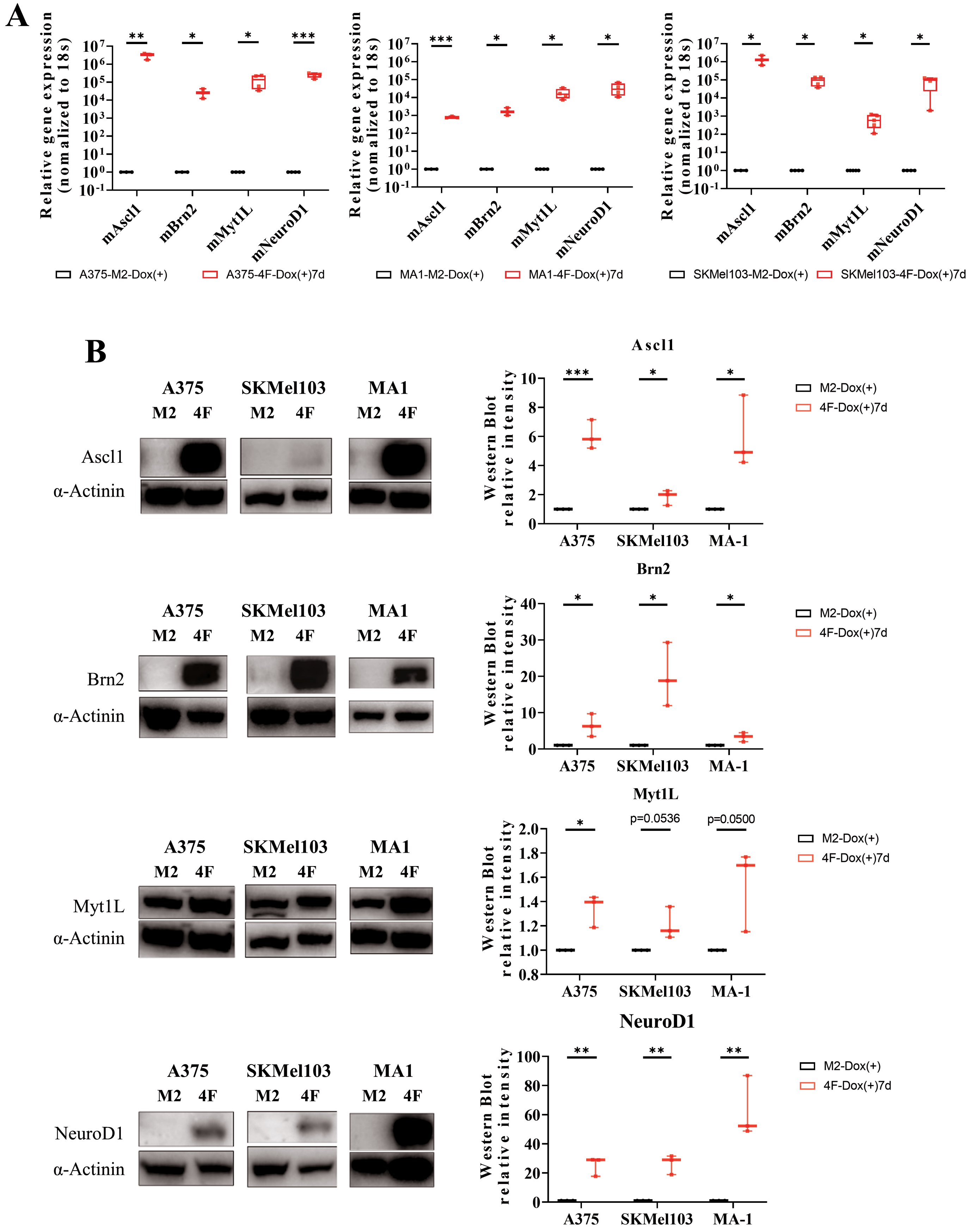
A
Relative gene expression (nomalized to 18s)
mAscl1
mBrn2
mMyt1L
mNeuroD1
A375-M2-Dox(+)
A375-4F-Dox(+)7d
MA1-M2-Dox(+)
MA1-4F-Dox(+)7d
SKMel103-M2-Dox(+)
SKMel103-4F-Dox(+)7d
B
A375
SKMel103
MA1
M2
4F
Ascl1
α-Actinin
Brn2
Myt1L
NeuroD1
Western Blot relative intensity
MA-1
M2-Dox(+)
4F-Dox(+)7d
p=0.0536
p=0.0500

Supplementary Figure 1. Expression of the transdifferentiation factors. A. Verification of the ectopic expression of the transdifferentiation factors in A375, MA1 and SKMel103 melanoma cells via qPCR. **B.** Verification of the ectopic expression of the four factors via western blot.

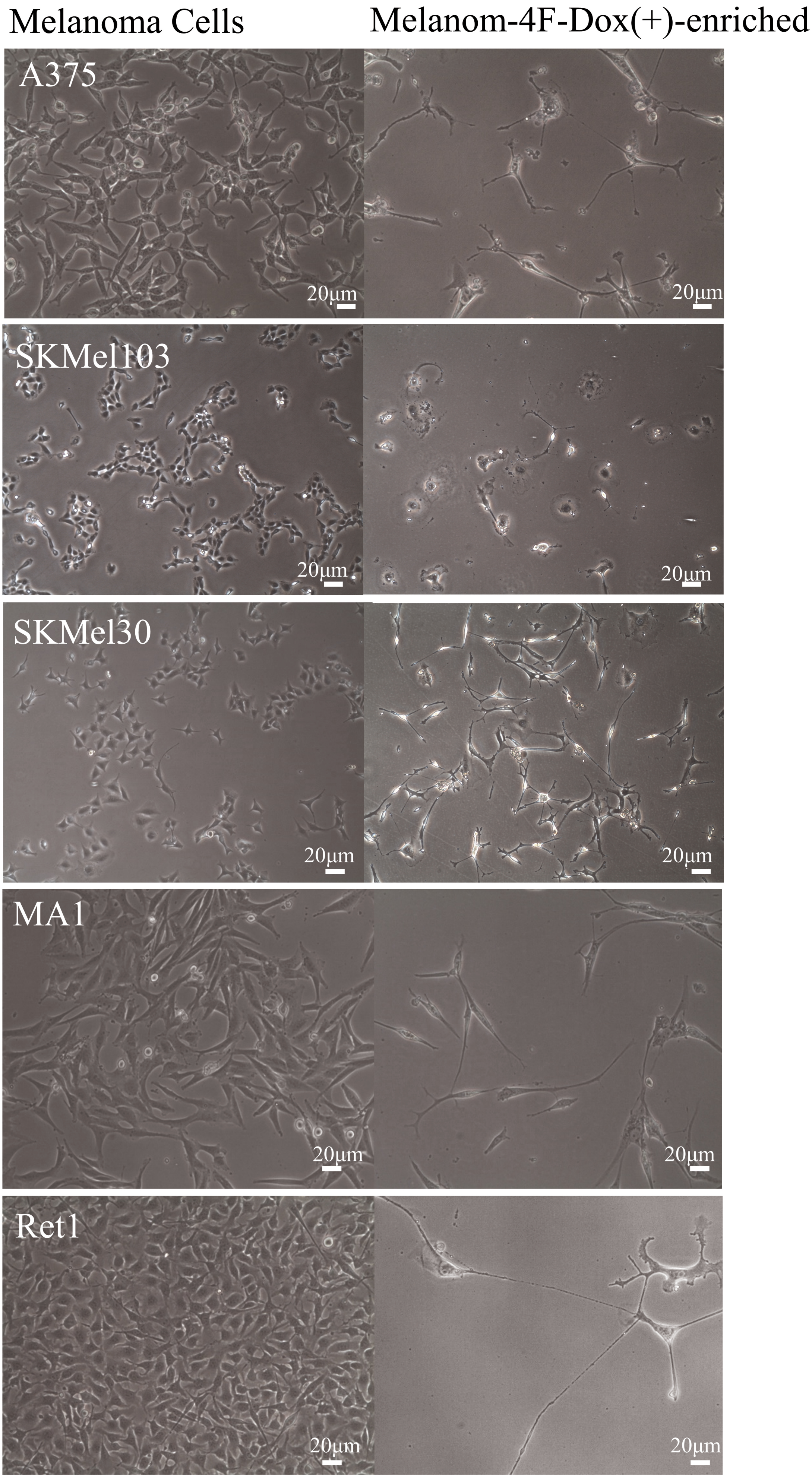

Melanoma Cells
Melanom-4F-Dox(+)-enriched
A375
SKMel103
SKMel30
MA1
Ret1
20μm

Supplementary Figure 2. Morphological changes in different cell lines. SKMel103 and SKMel30 are *NRAS*-mutated melanoma cell lines. MA-1 is a $BRAF^{V600E}$-mutated cell line from a 43-year-old male melanoma patient with resistance to targeted therapy. Ret1 is a mouse melanoma cell line. All cell lines show a completely different neuron-like morphology upon transdifferentiation.

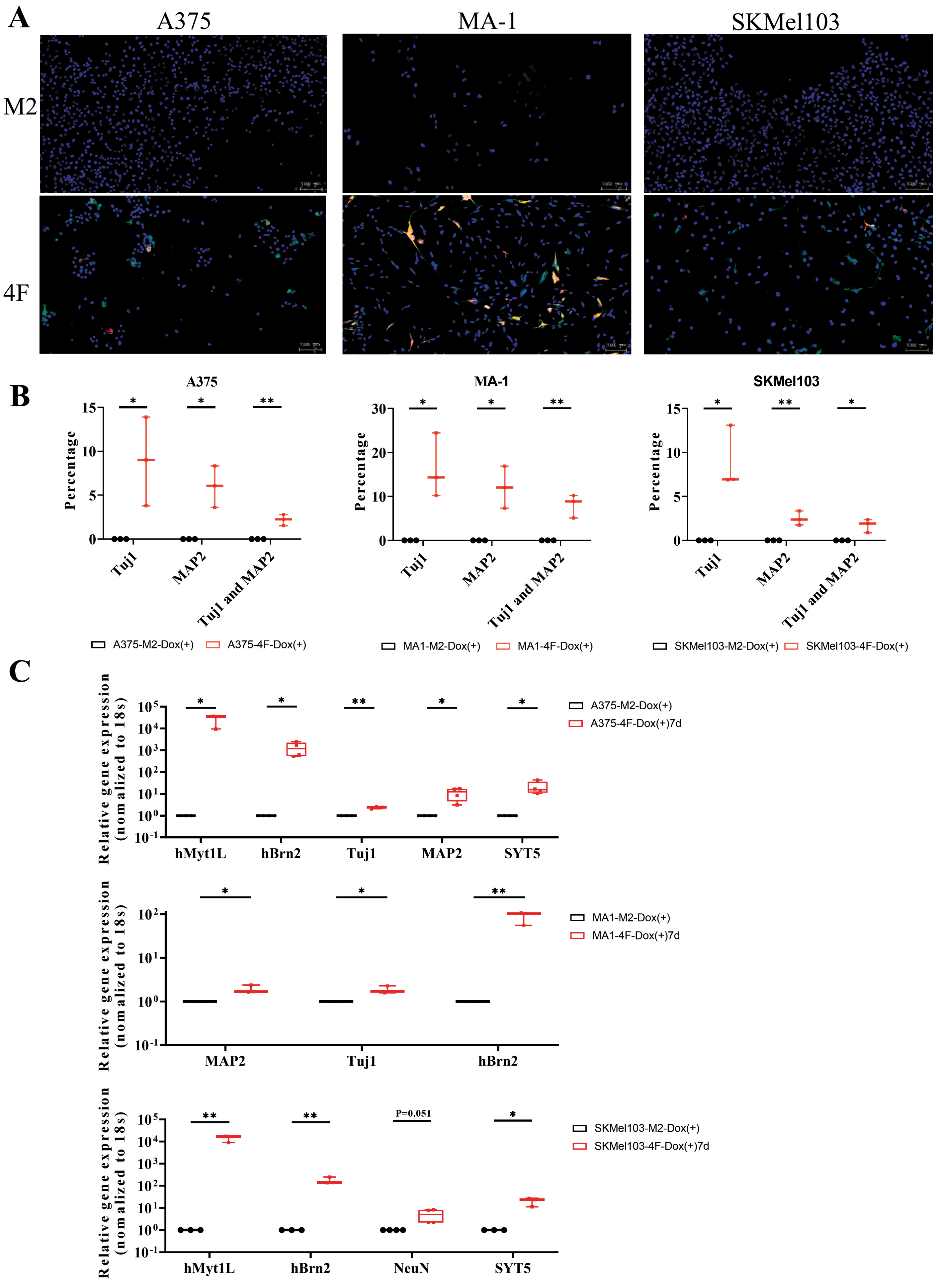
A
A375
MA-1
SKMel103
M2
4F
B
A375
MA-1
SKMel103
Percentage
Tuj1
MAP2
Tuj1 and MAP2
A375-M2-Dox(+)
A375-4F-Dox(+)
MA1-M2-Dox(+)
MA1-4F-Dox(+)
SKMel103-M2-Dox(+)
SKMel103-4F-Dox(+)
C
Relative gene expression (nomalized to 18s)
hMyt1L
hBrn2
Tuj1
MAP2
SYT5
A375-M2-Dox(+)
A375-4F-Dox(+)7d
MAP2
Tuj1
hBrn2
MA1-M2-Dox(+)
MA1-4F-Dox(+)7d
hMyt1L
hBrn2
NeuN
SYT5
P=0.051
SKMel103-M2-Dox(+)
SKMel103-4F-Dox(+)7d

Supplementary Figure 3. Elevation of the expression of neuronal markers in different melanoma cell lines upon transdifferentiation. A-B. Immunostaining revealed the expression of neuronal markers on the protein level in different melanoma cell lines 7 days after induction of transdifferentiation with doxycycline. DP refers to TUJ1/MAP2-double positive cells. **C.** The expression of neuronal markers could also be confirmed on the RNA level after induction with doxycycline for 7 days.

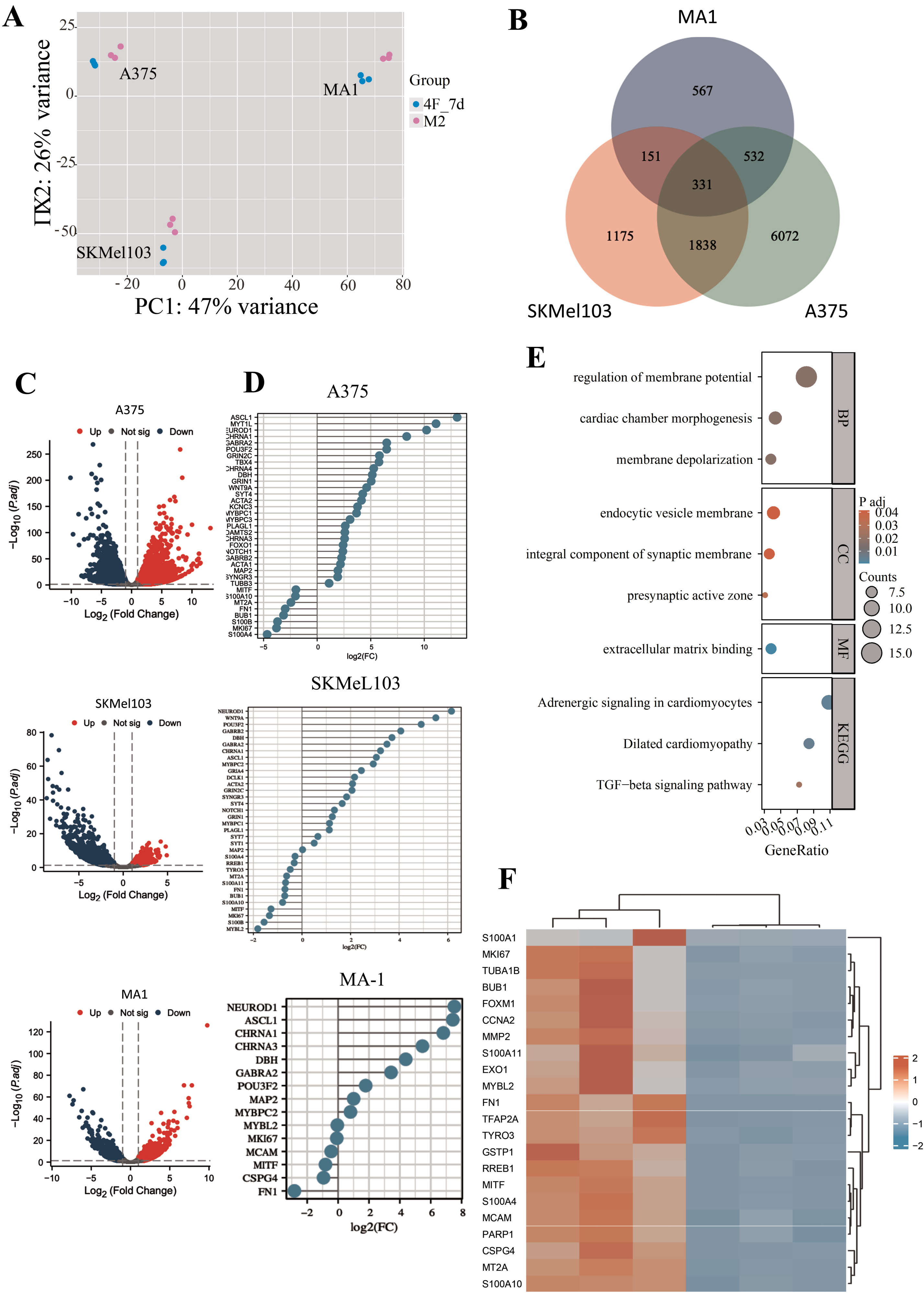
A
PC1: 47% variance
ПК2: 26% variance
A375
MA1
SKMel103
Group
4F_7d
M2
B
MA1
SKMel103
A375
567
151
532
331
1175
1838
6072
C
A375
SKMel103
MA1
Up
Not sig
Down
Log2 (Fold Change)
−Log10 (P.adj)
D
A375
SKMeL103
MA-1
log2(FC)
NEURODI
ASCL1
CHRNA1
CHRNA3
DBH
GABRA2
POU3F2
MAP2
MYBPC2
MYBL2
MKI67
MCAM
MITF
CSPG4
FN1
E
regulation of membrane potential
cardiac chamber morphogenesis
membrane depolarization
endocytic vesicle membrane
integral component of synaptic membrane
presynaptic active zone
extracellular matrix binding
Adrenergic signaling in cardiomyocytes
Dilated cardiomyopathy
TGF−beta signaling pathway
BP
CC
MF
KEGG
P adj
Counts
GeneRatio
F
S100A1
MKI67
TUBA1B
BUB1
FOXM1
CCNA2
MMP2
S100A11
EXO1
MYBL2
FN1
TFAP2A
TYRO3
GSTP1
RREB1
MITF
S100A4
MCAM
PARP1
CSPG4
MT2A
S100A10

Supplementary Figure 4. RNAseq analysis in different cell lines. A. PCA graph for different cell lines and groups. **B.** Venn diagram of DEGs in the melanoma-M2 and melanoma-4F groups of A375, SKMel103, and MA1 cell lines. **C.** Volcano plot of differentially expressed genes in A375, SKMel103 and MA1 cells. **D.** Fold change of expression of different melanoma and neuronal markers in 3 melanoma cell lines upon transdifferentiation. **E.** Results of GO-KEGG analysis based on the 215 DEGs. **F.** RNAseq revealed a downregulation of melanoma markers (left 3 columns: A375-M2, right 3 columns: A375-4F) 7 days upon induction of transdifferentiation with doxycycline.

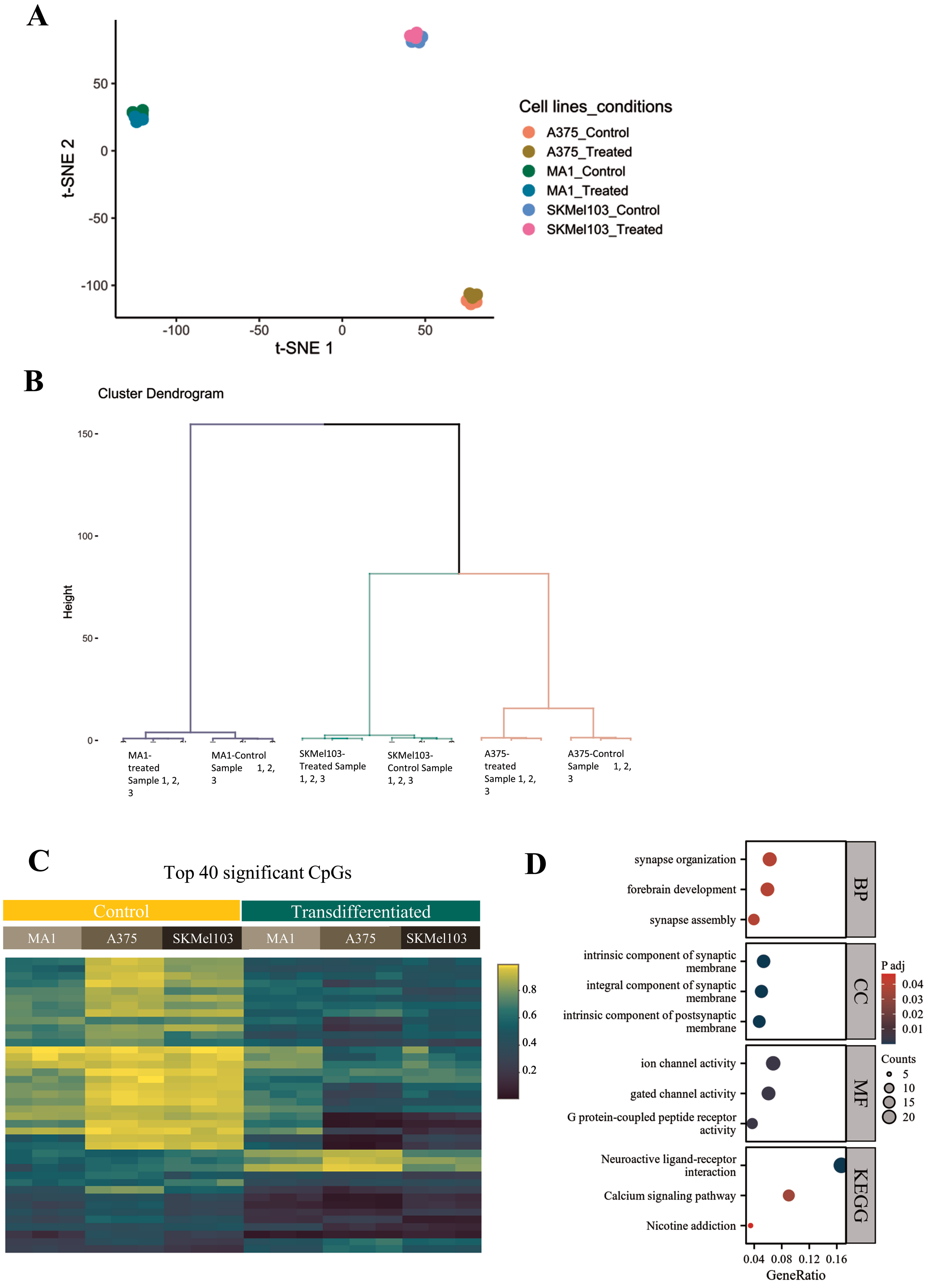
A
t-SNE 2
t-SNE 1
Cell lines_conditions
A375_Control
A375_Treated
MA1_Control
MA1_Treated
SKMel103_Control
SKMel103_Treated
B
Cluster Dendrogram
Height
MA1-treated Sample 1, 2, 3
MA1-Control Sample 1, 2, 3
SKMel103-Treated Sample 1, 2, 3
SKMel103-Control Sample 1, 2, 3
A375-treated Sample 1, 2, 3
A375-Control Sample 1, 2, 3
C
Top 40 significant CpGs
Control
Transdifferentiated
MA1
A375
SKMel103
MA1
A375
SKMel103
D
synapse organization
forebrain development
synapse assembly
intrinsic component of synaptic membrane
integral component of synaptic membrane
intrinsic component of postsynaptic membrane
ion channel activity
gated channel activity
G protein-coupled peptide receptor activity
Neuroactive ligand-receptor interaction
Calcium signaling pathway
Nicotine addiction
BP
CC
MF
KEGG
P adj
Counts
GeneRatio

Supplementary Figure 5. DNA methylation analysis in different cell lines. A. PCA graph reveals that the transdifferentiated (treated) cells shift upwards. **B.** In the cluster dendrogram each cell line stays in their cluster, while 4F cells and M2 cells are in separate subclusters. **C.** Heatmap of top 40 significantly methylated CpGs in 3 cell lines. **D.** Results of GO-KEGG analysis based on differentially methylated genes in A375 cells (comparison between A375-M2 and A375-4F).

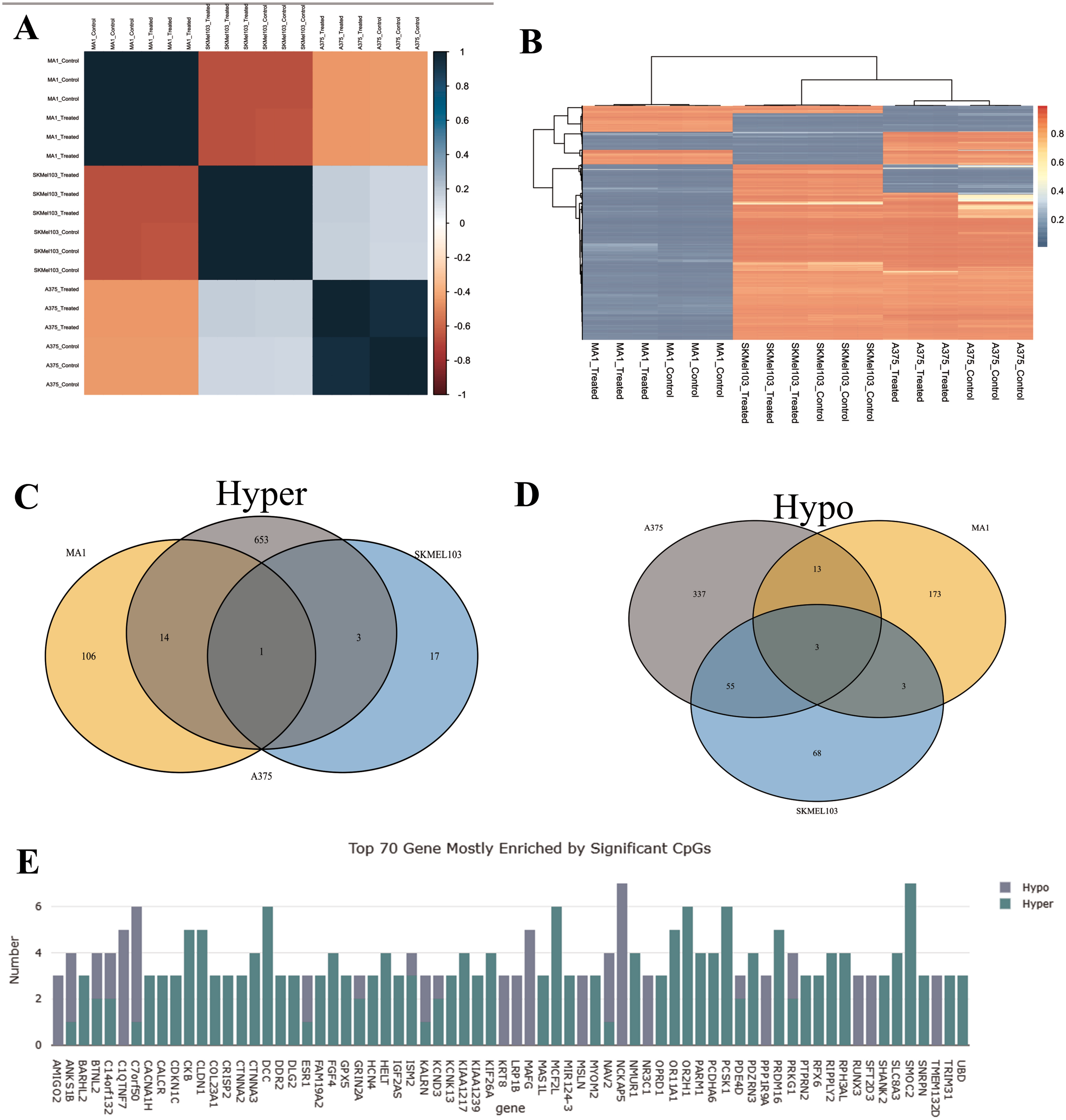

A
MA1_Control
MA1_Treated
SKMel103_Treated
SKMel103_Control
A375_Treated
A375_Control
1
0.8
0.6
0.4
0.2
0
-0.2
-0.4
-0.6
-0.8
-1
B
0.8
0.6
0.4
0.2
C
Hyper
MA1
SKMEL103
A375
653
106
14
1
3
17
D
Hypo
A375
MA1
SKMEL103
337
13
173
3
55
3
68
E
Top 70 Gene Mostly Enriched by Significant CpGs
Hypo
Hyper
Number
gene
AMIGO2
ANKS1B
BARHL2
BTNL2
C14orf132
C1QTNF7
C7orf50
CACNA1H
CALCR
CDKN1C
CKB
CLDN1
COL23A1
CRISP2
CTNNA2
CTNNA3
DCC
DDR2
DLG2
ESR1
FAM19A2
FGF4
GPX5
GRIN2A
HCN4
HELT
IGF2AS
ISM2
KALRN
KCND3
KCNK13
KIAA1217
KIAA1239
KIF26A
KRT8
LRP1B
MAFG
MAS1L
MCF2L
MIR124-3
MSLN
MYOM2
NAV2
NCKAP5
NMUR1
NR3C1
OPRD1
OR1A1
OR2H1
PARM1
PCDHA6
PCSK1
PDE4D
PDZRN3
PPP1R9A
PRDM16
PRKG1
PTPRN2
RFX6
RIPPLY2
RPH3AL
RUNX3
SFT2D3
SHANK2
SLC8A3
SMOC2
SNRPN
TMEM132D
TRIM31
UBD

Supplementary Figure 6. Extended DNA methylation analysis in different cell lines. A. The correlation plot shows that the A375 cells have the most DNA methylation alterations, while the least alterations were detected in MA1, and SKMel103. **B.** Overview of top 5000 variable CpGs in each control group and their methylation status after transdifferentiation. **C-D.** Venn diagram about the common hyper- or hypomethylated genes in 3 melanoma cell lines. **E.** The top enriched genes according to significant hypo- or hypermethylated CpGs in A375 melanoma cells.

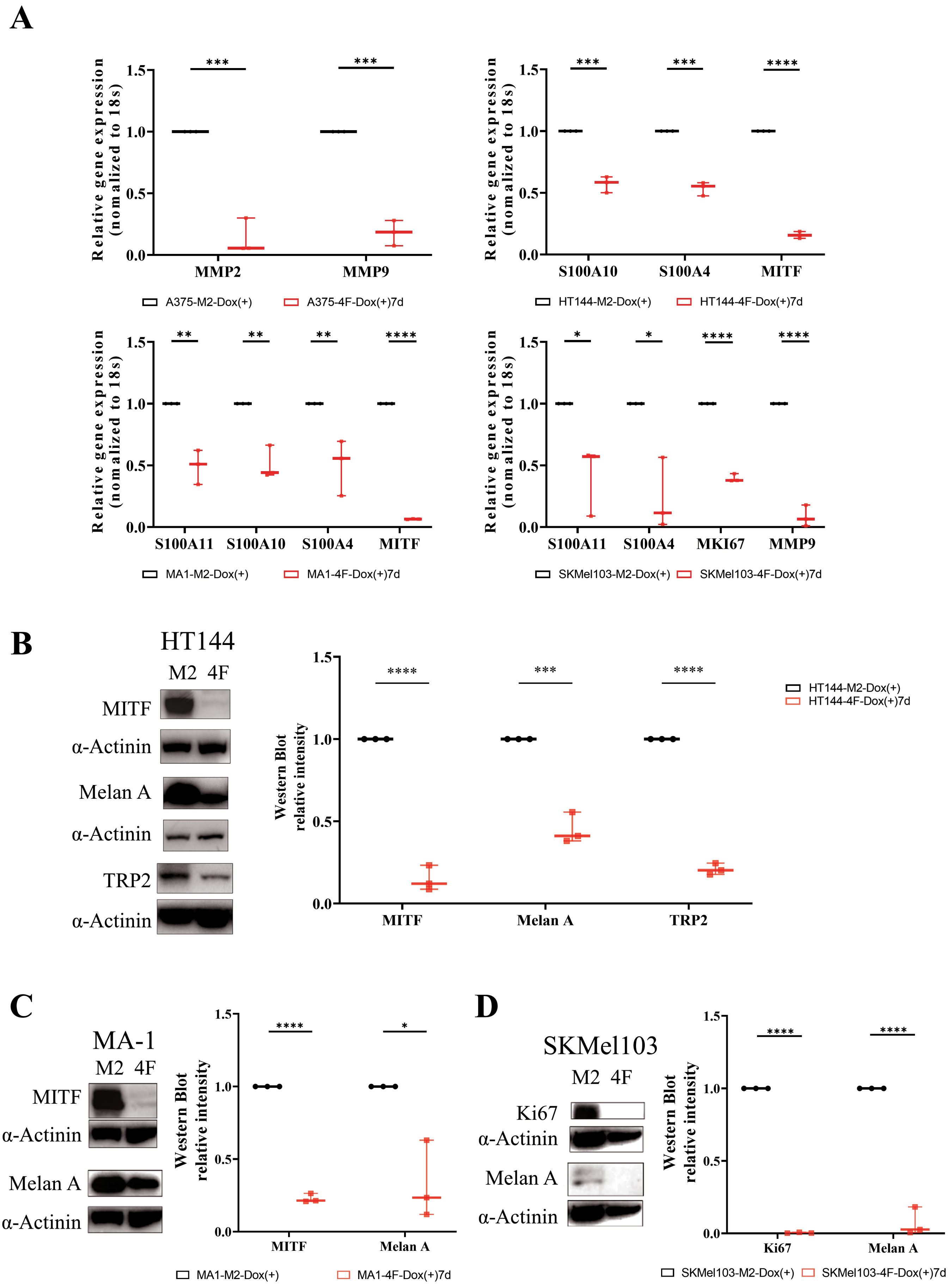

A
Relative gene expression (nomalized to 18s)
1.5
1.0
0.5
0.0
***
***
MMP2
MMP9
A375-M2-Dox(+)
A375-4F-Dox(+)7d
***
***
****
S100A10
S100A4
MITF
HT144-M2-Dox(+)
HT144-4F-Dox(+)7d
**
**
**
****
S100A11
S100A10
S100A4
MITF
MA1-M2-Dox(+)
MA1-4F-Dox(+)7d
*
*
****
****
S100A11
S100A4
MKI67
MMP9
SKMel103-M2-Dox(+)
SKMel103-4F-Dox(+)7d
B
HT144
M2 4F
MITF
α-Actinin
Melan A
α-Actinin
TRP2
α-Actinin
Western Blot relative intensity
****
***
****
MITF
Melan A
TRP2
HT144-M2-Dox(+)
HT144-4F-Dox(+)7d
C
MA-1
M2 4F
MITF
α-Actinin
Melan A
α-Actinin
Western Blot relative intensity
****
*
MITF
Melan A
MA1-M2-Dox(+)
MA1-4F-Dox(+)7d
D
SKMel103
M2 4F
Ki67
α-Actinin
Melan A
α-Actinin
Western Blot relative intensity
****
****
Ki67
Melan A
SKMel103-M2-Dox(+)
SKMel103-4F-Dox(+)7d

Supplementary Figure 7. Markers related to melanoma, tumor proliferation and invasion are downregulated after transdifferentiation. A. qPCR analysis showing a downregulation of melanoma markers in A375, HT144, MA1 and SKMel103 melanoma cells 7 days upon induction of transdifferentiation with doxycycline. **B.** Melanoma markers were downregulated at the protein level 7 days upon induction of transdifferentiation with doxycycline. **C-D.** Melanoma markers were also downregulated at the protein level upon treatment with doxycycline for 7 days.

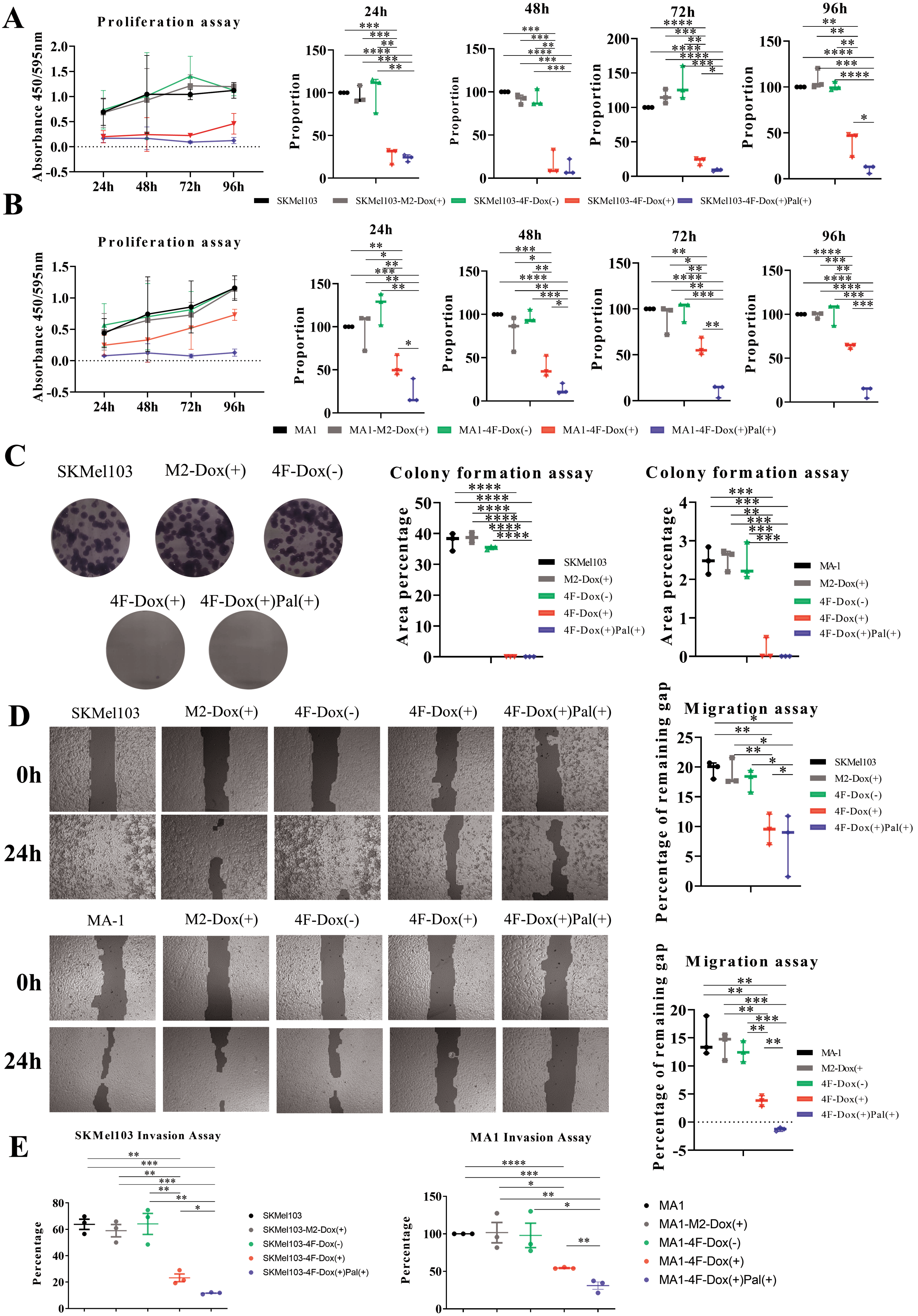
A
Proliferation assay
Absorbance 450/595nm
24h
48h
72h
96h
Proportion
SKMel103
SKMel103-M2-Dox(+)
SKMel103-4F-Dox(-)
SKMel103-4F-Dox(+)
SKMel103-4F-Dox(+)Pal(+)
B
Proliferation assay
Absorbance 450/595nm
MA1
MA1-M2-Dox(+)
MA1-4F-Dox(-)
MA1-4F-Dox(+)
MA1-4F-Dox(+)Pal(+)
C
SKMel103
M2-Dox(+)
4F-Dox(-)
4F-Dox(+)
4F-Dox(+)Pal(+)
Colony formation assay
Area percentage
MA-1
D
0h
24h
MA-1
Migration assay
Percentage of remaining gap
E
SKMel103 Invasion Assay
MA1 Invasion Assay
Percentage

Supplementary Figure 8. Loss of tumor cell properties of transdifferentiated MA1 and SKMel103 cells *in vitro*. A-B. Transdifferentiated melanoma cells exhibited reduced proliferation capacity compared to their M2-vector counterparts. Transdifferentiated cells after enrichment exhibited almost no proliferation capacity. **C.** Colony formation assay revealed that transdifferentiated-4F cells were much less tumorigenic, while transdifferentiated and enriched 4F-cells showed no tumorigenicity. SKMel103 cells were treated with doxycycline for 6 days followed by 4 days without doxycycline. MA1 cells were treated with doxycycline for 10 days followed by 4 days without doxycycline. **D.** Migration capacity was markedly reduced in the transdifferentiated group and even more reduced in the transdifferentiated and enriched group. **E.** Invasion capacity was significantly reduced upon transdifferentiation and palbociclib treatment.

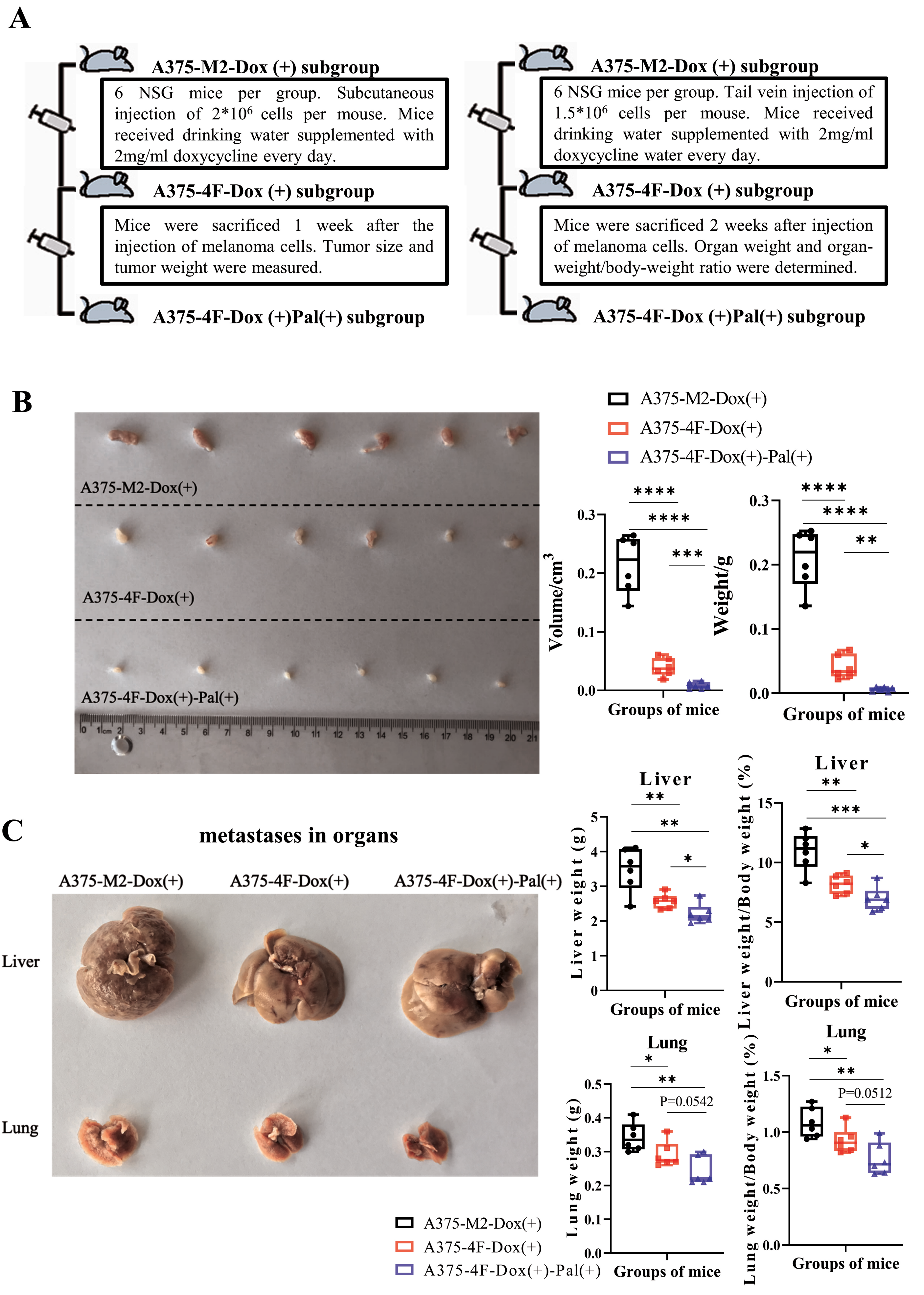
A
A375-M2-Dox (+) subgroup
6 NSG mice per group. Subcutaneous injection of 2*10⁶ cells per mouse. Mice received drinking water supplemented with 2mg/ml doxycycline every day.
A375-4F-Dox (+) subgroup
Mice were sacrificed 1 week after the injection of melanoma cells. Tumor size and tumor weight were measured.
A375-4F-Dox (+)Pal(+) subgroup
A375-M2-Dox (+) subgroup
6 NSG mice per group. Tail vein injection of 1.5*10⁶ cells per mouse. Mice received drinking water supplemented with 2mg/ml doxycycline water every day.
A375-4F-Dox (+) subgroup
Mice were sacrificed 2 weeks after injection of melanoma cells. Organ weight and organ-weight/body-weight ratio were determined.
A375-4F-Dox (+)Pal(+) subgroup
B
A375-M2-Dox(+)
A375-4F-Dox(+)
A375-4F-Dox(+)-Pal(+)
Volume/cm3
Weight/g
Groups of mice
Groups of mice
C
metastases in organs
A375-M2-Dox(+)
A375-4F-Dox(+)
A375-4F-Dox(+)-Pal(+)
Liver
Lung
Liver
Liver weight (g)
Groups of mice
Liver
Liver weight/Body weight (%)
Groups of mice
Lung
Lung weight (g)
P=0.0542
Groups of mice
Lung
Lung weight/Body weight (%)
P=0.0512
Groups of mice
A375-M2-Dox(+)
A375-4F-Dox(+)
A375-4F-Dox(+)-Pal(+)

Supplementary Figure 9. Transdifferentiated A375 cells show reduced tumorigenic and metastastatic capacity *in vivo*. A. Schematic overview of the *in vivo* experimental procedure. **B.** The size and weight of tumors derived from parental melanoma cells upon subcutaneous injection into NSG mice were significantly higher compared to tumors derived from transdifferentiated cells. Almost no tumor was formed upon injection of transdifferentiated and enriched cells. **C.** Measurement of lung and liver weight upon intravenous injection of transdifferentiated or parental melanoma cells into NSG mice. Both lung and liver weight were significantly lower upon injection of transdifferentiated cells. Moreover, metastases were visible especially in the liver after injection of parental melanoma cells but not after injection of transdifferentiated cells.

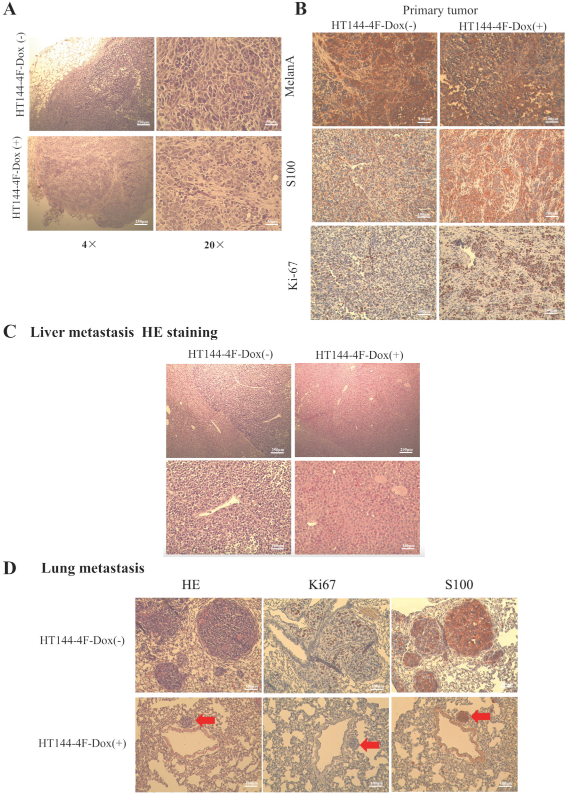
A
HT144-4F-Dox (-)
HT144-4F-Dox (+)
250μm
50μm
4×
20×
B
Primary tumor
HT144-4F-Dox(-)
HT144-4F-Dox(+)
MelanA
S100
Ki-67
100μm
C
Liver metastasis HE staining
HT144-4F-Dox(-)
HT144-4F-Dox(+)
250μm
100μm
D
Lung metastasis
HE
Ki67
S100
HT144-4F-Dox(-)
HT144-4F-Dox(+)
100μm

Supplementary Figure 10. Histological analysis of tumors derived from parental or transdifferentiated melanoma cells. A. In the Dox (-) group, tumor cells infiltrated extensively, reaching deep into the adipose tissue, exhibiting significant atypia, and distributed densely. In contrast, in the Dox (+) group, tumor cells were confined and did not breach the capsule, resulting in a relatively loose arrangement. **B.** Both induced and non-induced cells showed strong melanocytic properties, suggesting that 4F-Dox (+) cells without enrichment contain melanoma-like cells, which dominate the tumor formation *in vivo*. **C.** No metastases were found in the liver in Dox (+) group. **D.** Of all mice lung slices, there was only one metastasis detectable, which showed very low expression of the proliferation marker Ki67.

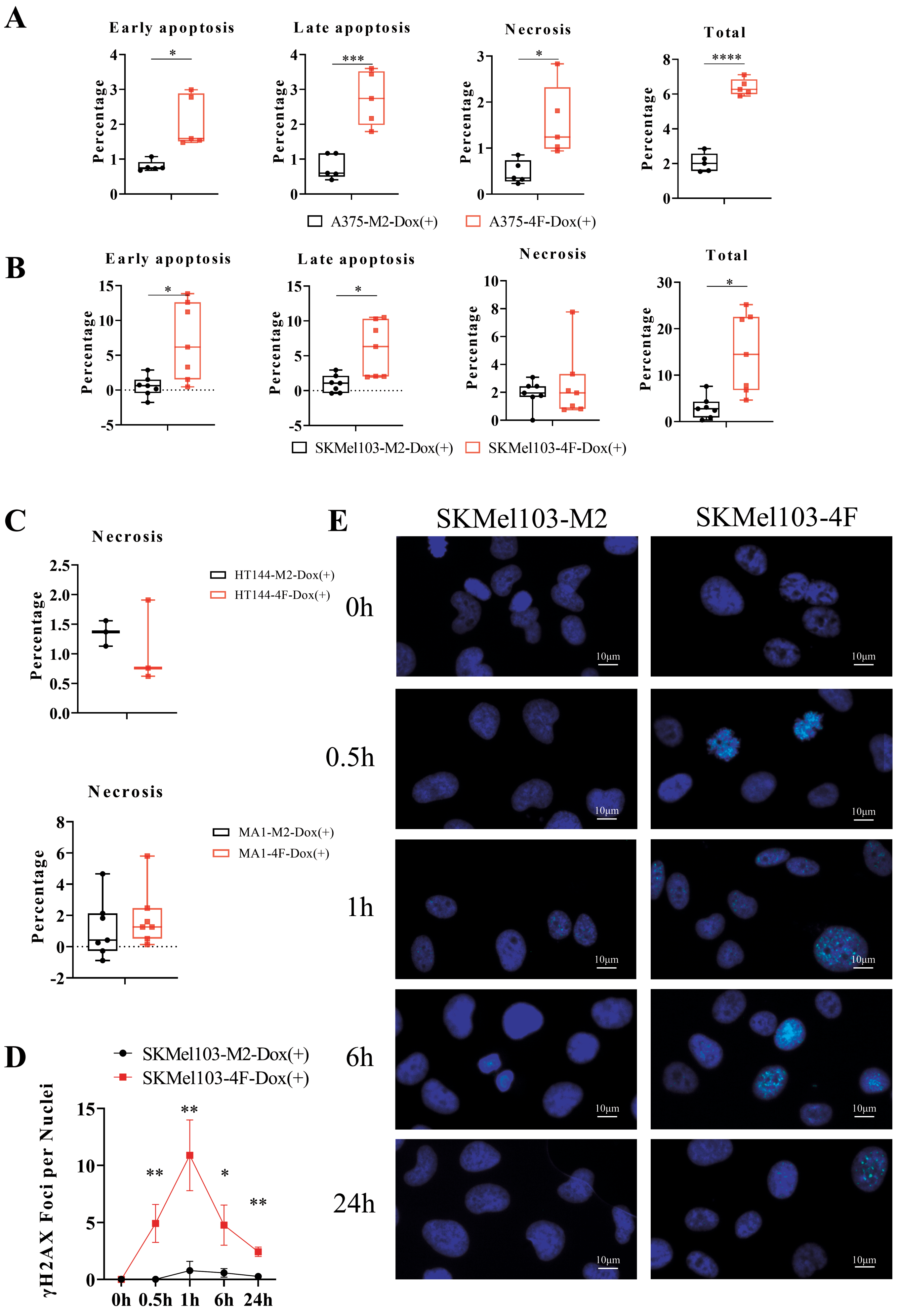
A
Early apoptosis
Late apoptosis
Necrosis
Total
Percentage
A375-M2-Dox(+)
A375-4F-Dox(+)
B
Early apoptosis
Late apoptosis
Necrosis
Total
Percentage
SKMel103-M2-Dox(+)
SKMel103-4F-Dox(+)
C
Necrosis
Percentage
HT144-M2-Dox(+)
HT144-4F-Dox(+)
Necrosis
Percentage
MA1-M2-Dox(+)
MA1-4F-Dox(+)
D
SKMel103-M2-Dox(+)
SKMel103-4F-Dox(+)
γH2AX Foci per Nuclei
0h 0.5h 1h 6h 24h
E
SKMel103-M2
SKMel103-4F
0h
0.5h
1h
6h
24h
10μm

Supplementary Figure 11. Increased sensitivity of transdifferentiated cells to radiotherapy. A-C. Quantification of the percentage of apoptotic cells 96h after treatment with 3Gy radiation. Early apoptotic cells (Annexin V^{+}/$DAPI^{-}$) appear in the lower right quadrant, indicating phosphatidylserine externalization with an intact membrane. Late apoptotic cells (Annexin V^{+}/$DAPI^{+}$) are in the upper right quadrant, characterized by both phosphatidylserine exposure and membrane permeability. Necroptotic cells (Annexin V^{-}/$DAPI^{+}$) are in the upper left quadrant, showing membrane rupture without phosphatidylserine externalization. The percentage of apoptotic and dead cells was much higher among the transdifferentiated cells in comparison to the parental melanoma cells. This was true for the cell lines MA1, SKMel103 and A375. **D-E.** gH2AX analysis 0.5, 1, 6, or 24h after single treatment of MA1 and SKMel103 cells with 3Gy radiation.
