# Supplementary material for: Direct transdifferentiation of tumorigenic melanoma cells induces tumor cell reversion
Source: Cell Death Dis. 2025 Jul 25;16(1):563. doi: 10.1038/s41419-025-07863-y (PMC12297470; doi:10.1038/s41419-025-07863-y)
Supplement: Supplementary file 2 — western blots [file 41419_2025_7863_MOESM2_ESM.pptx]

## Slide 1
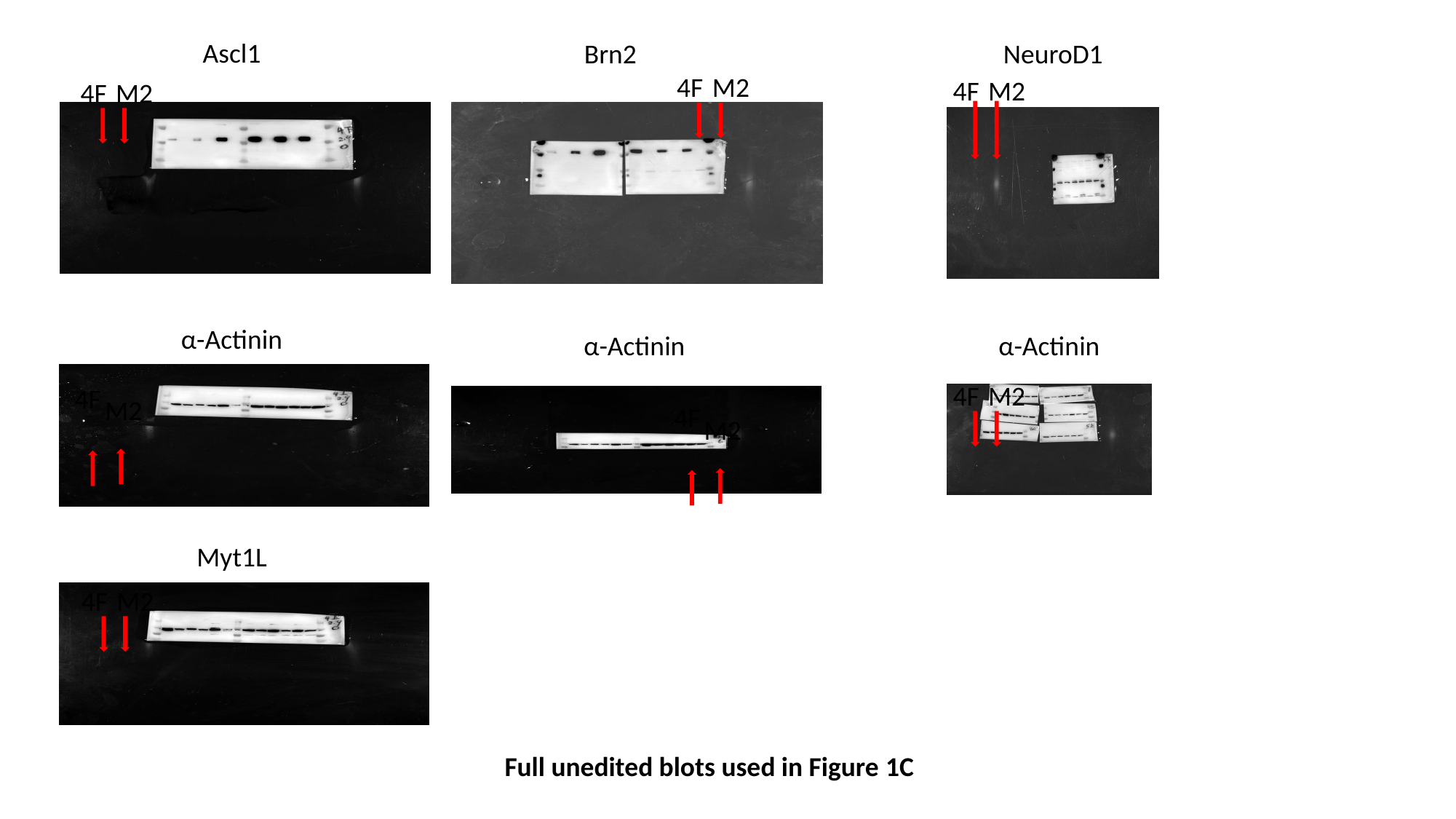

Ascl1
Brn2
NeuroD1
4F
M2
4F
M2
4F
M2
α-Actinin
α-Actinin
α-Actinin
4F
M2
4F
M2
4F
M2
Myt1L
4F
M2
Full unedited blots used in Figure 1C

## Slide 2
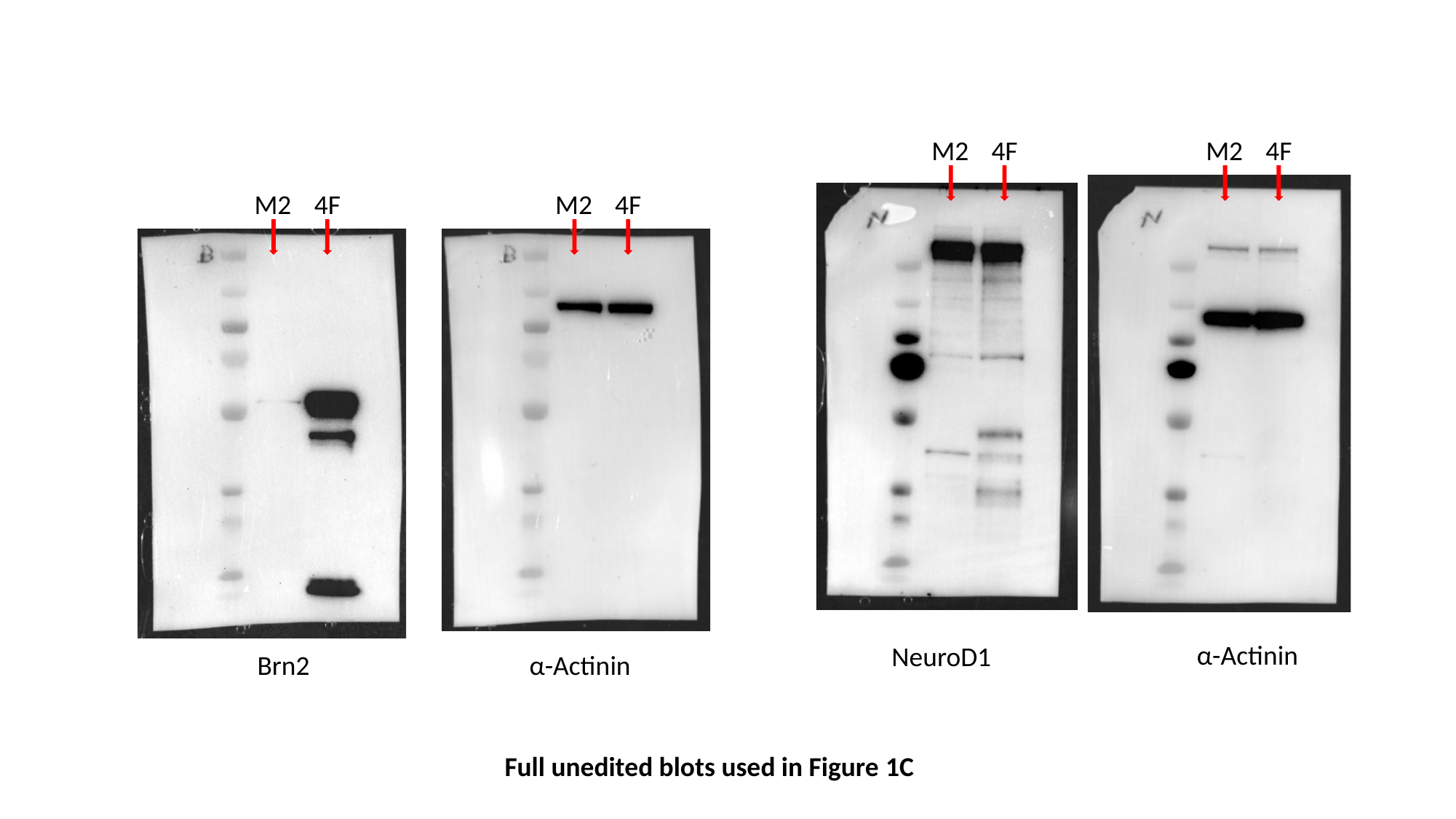

M2
4F
M2
4F
M2
4F
M2
4F
α-Actinin
NeuroD1
Brn2
α-Actinin
Full unedited blots used in Figure 1C

## Slide 3
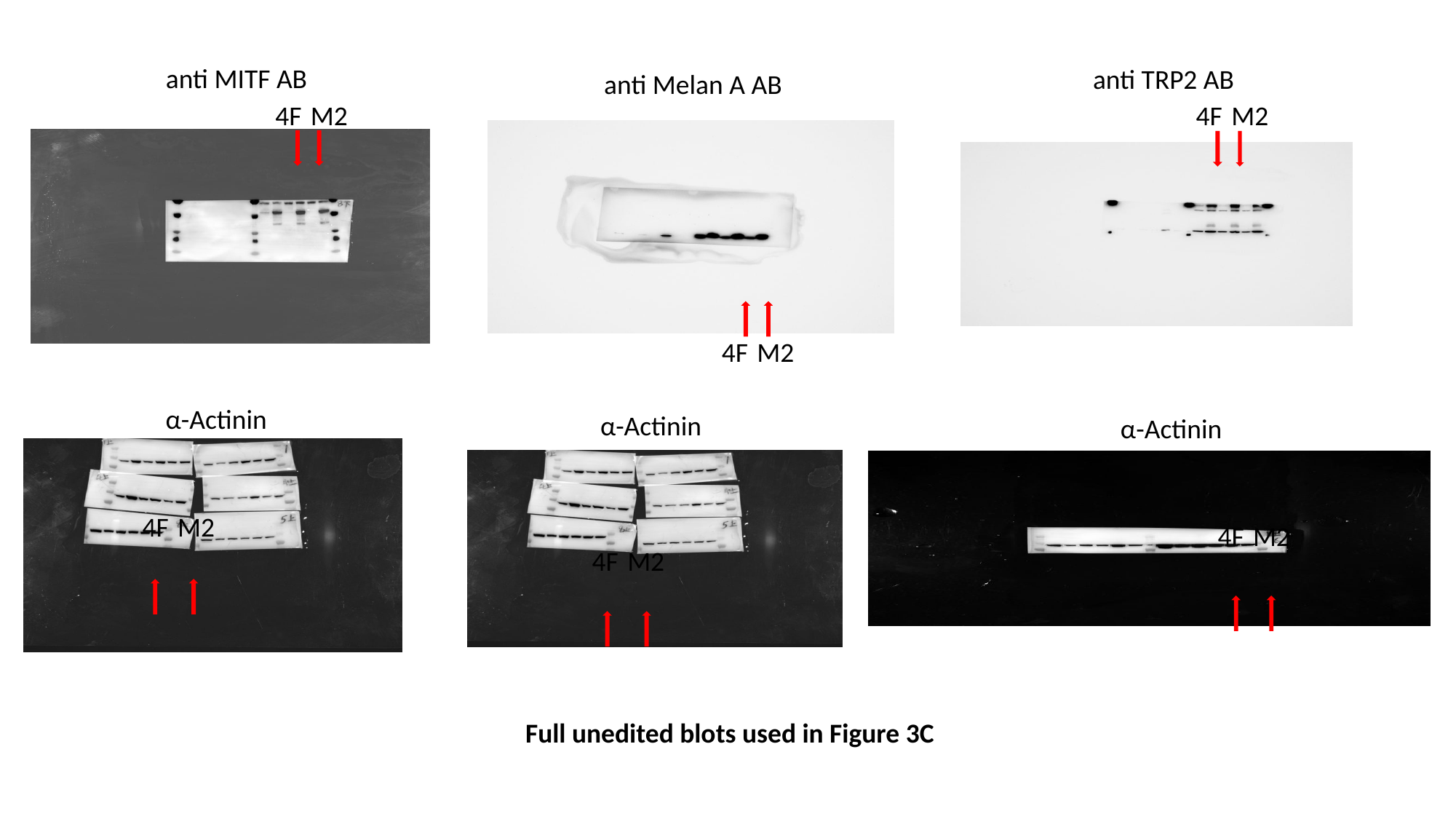

anti MITF AB
anti TRP2 AB
anti Melan A AB
4F
M2
4F
M2
4F
M2
α-Actinin
α-Actinin
α-Actinin
4F
M2
4F
M2
4F
M2
Full unedited blots used in Figure 3C

## Slide 4
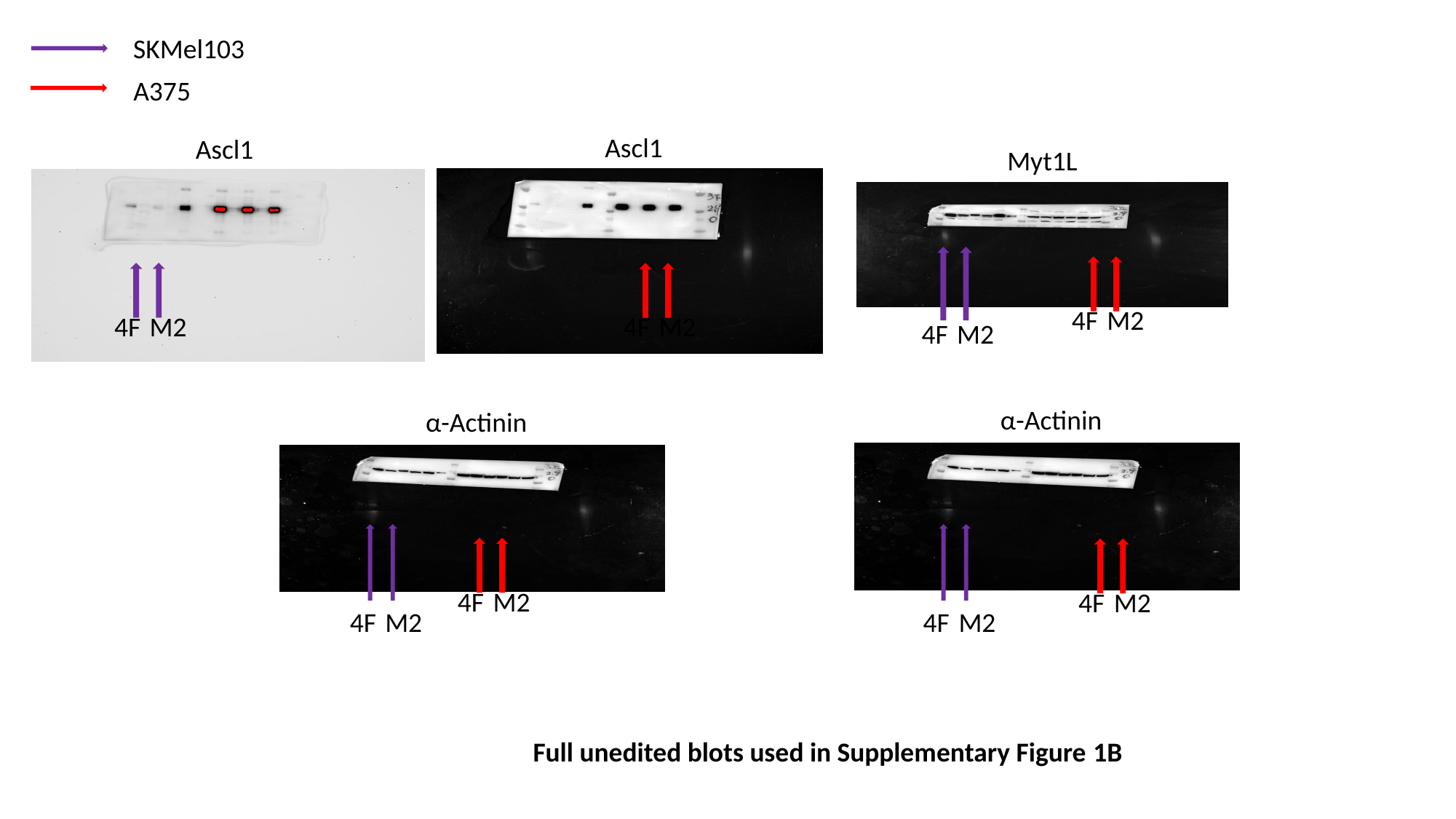

SKMel103
A375
Ascl1
Ascl1
Myt1L
4F
M2
4F
M2
4F
M2
4F
M2
α-Actinin
α-Actinin
4F
M2
4F
M2
4F
M2
4F
M2
Full unedited blots used in Supplementary Figure 1B

## Slide 5
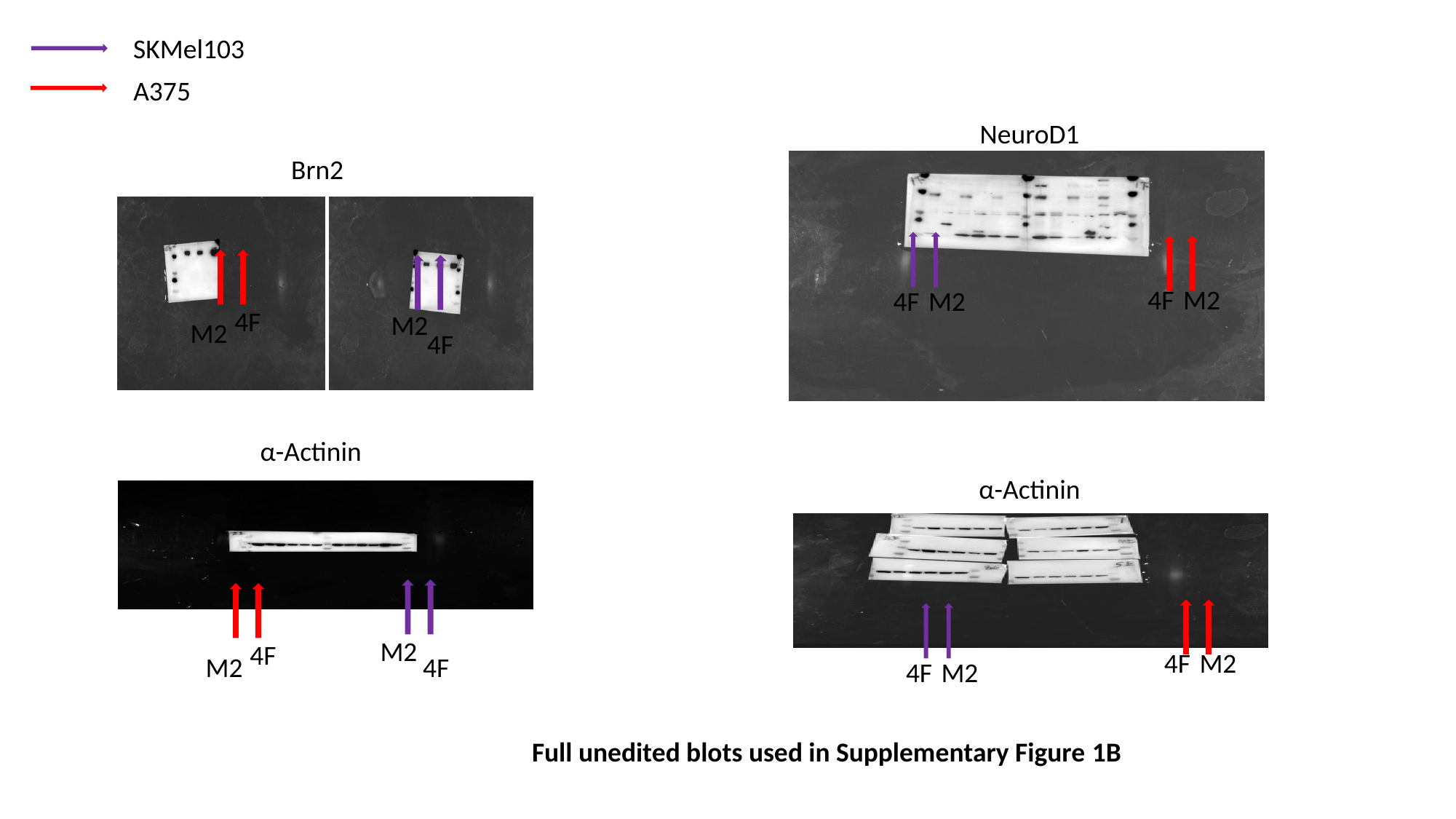

SKMel103
A375
NeuroD1
Brn2
4F
M2
4F
M2
4F
M2
M2
4F
α-Actinin
α-Actinin
M2
4F
4F
M2
M2
4F
4F
M2
Full unedited blots used in Supplementary Figure 1B

## Slide 6
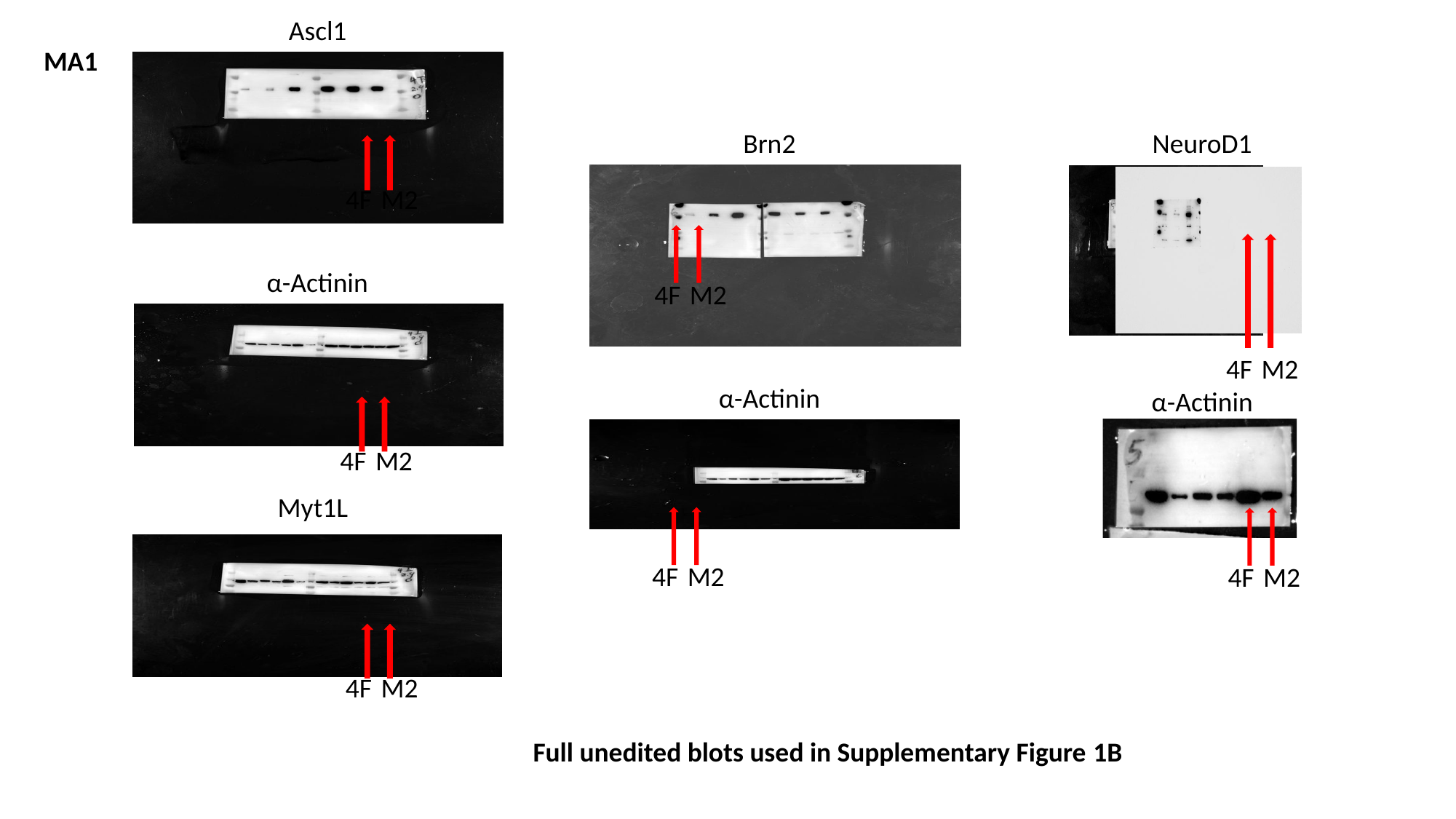

Ascl1
MA1
Brn2
NeuroD1
4F
M2
α-Actinin
4F
M2
4F
M2
α-Actinin
α-Actinin
4F
M2
Myt1L
4F
M2
4F
M2
4F
M2
Full unedited blots used in Supplementary Figure 1B

## Slide 7
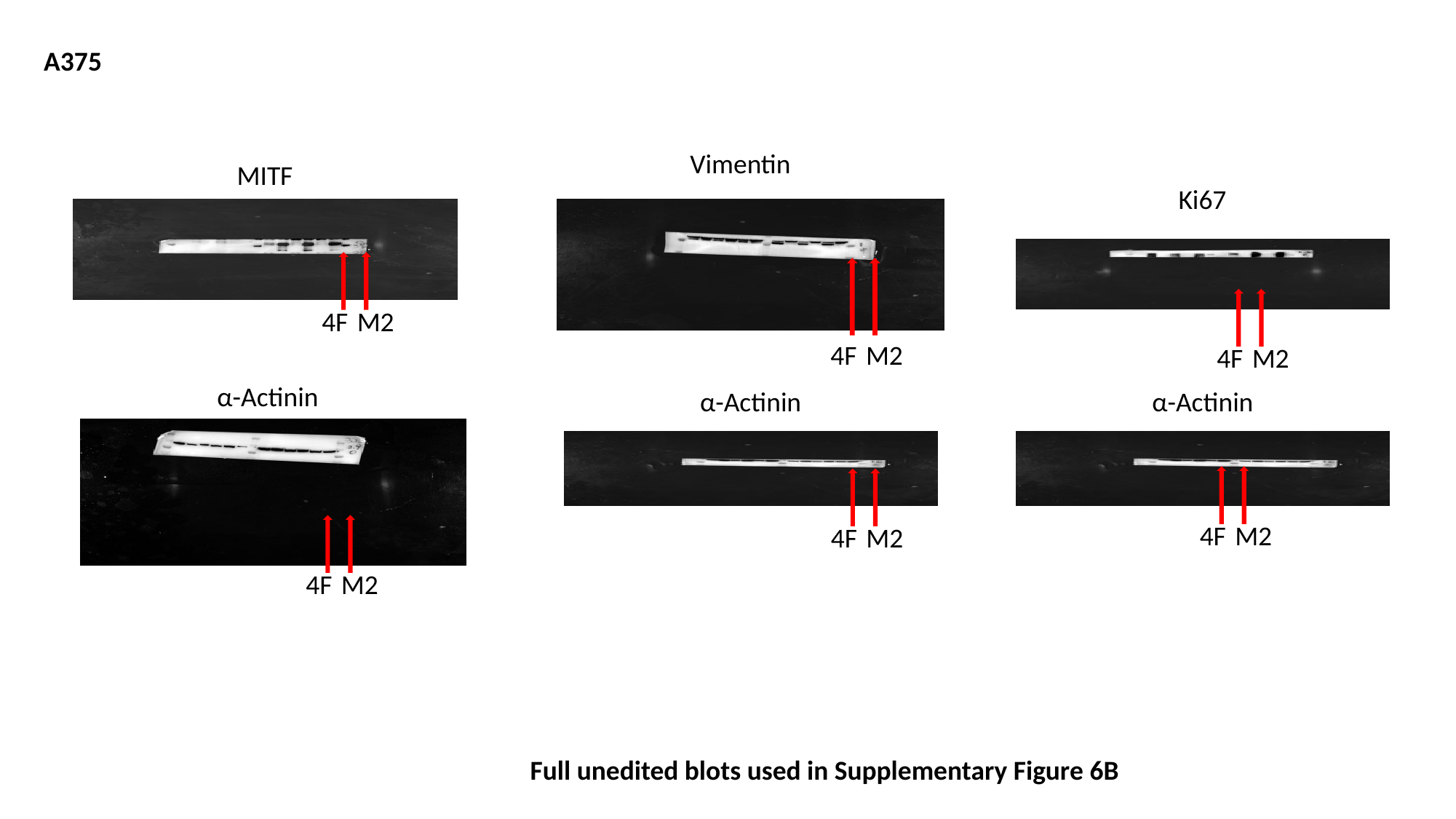

A375
Vimentin
MITF
Ki67
4F
M2
4F
M2
4F
M2
α-Actinin
α-Actinin
α-Actinin
4F
M2
4F
M2
4F
M2
Full unedited blots used in Supplementary Figure 6B

## Slide 8
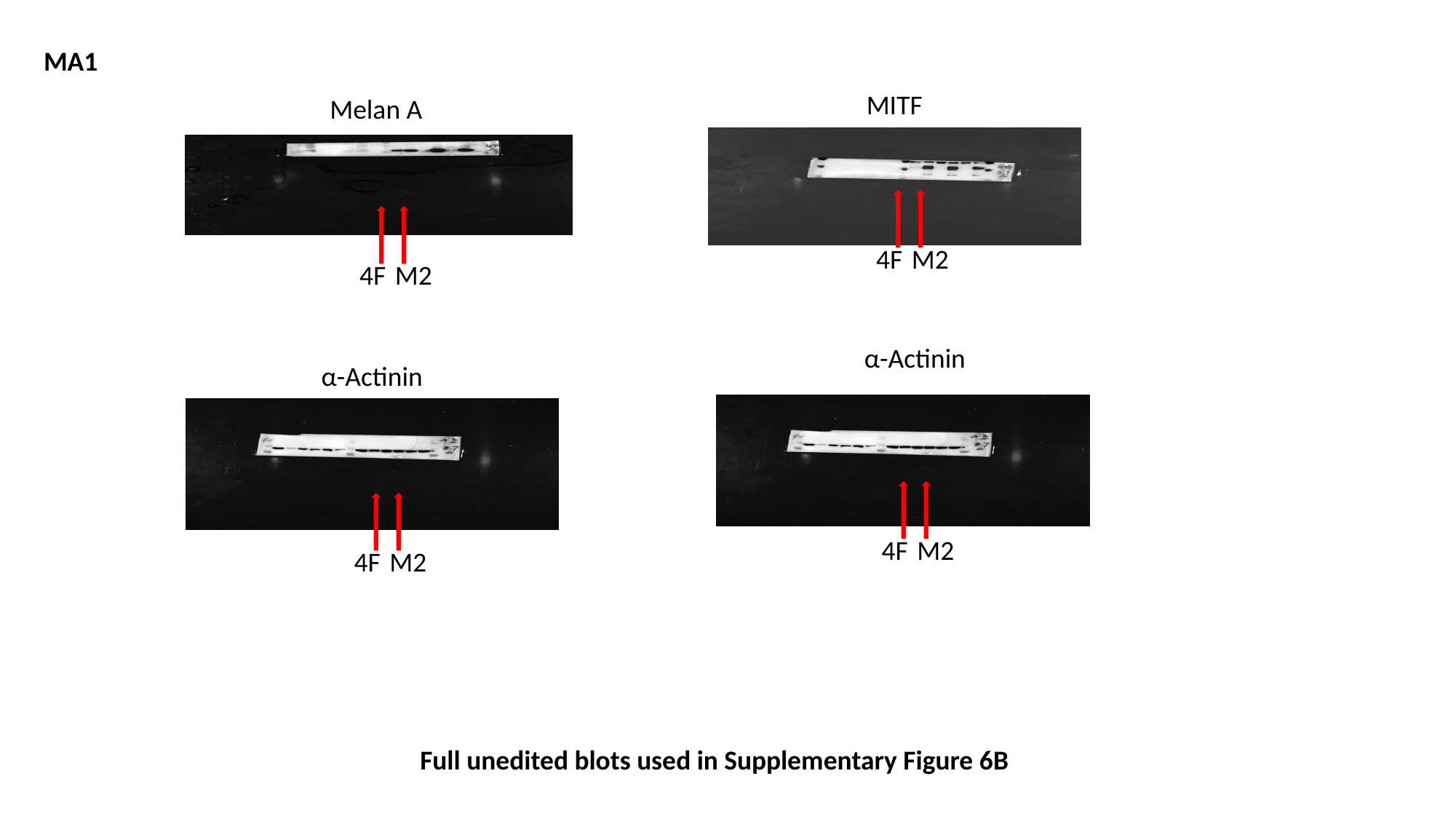

MA1
MITF
Melan A
4F
M2
4F
M2
α-Actinin
α-Actinin
4F
M2
4F
M2
Full unedited blots used in Supplementary Figure 6B

## Slide 9
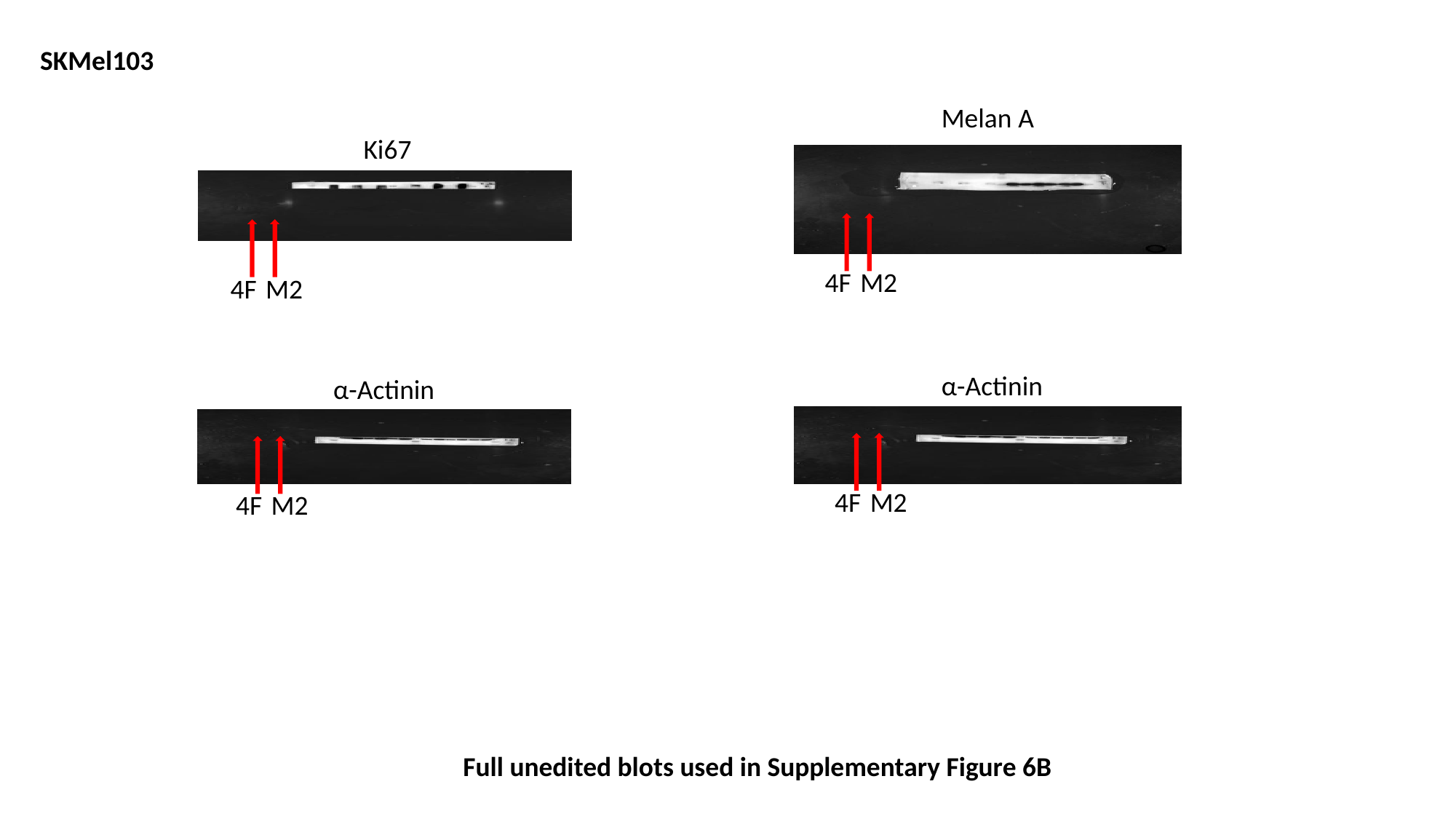

SKMel103
Melan A
Ki67
4F
M2
4F
M2
α-Actinin
α-Actinin
4F
M2
4F
M2
Full unedited blots used in Supplementary Figure 6B
