# Supplementary material for: Direct transdifferentiation of tumorigenic melanoma cells induces tumor cell reversion
Source: Cell Death Dis. 2025 Jul 25;16(1):563. doi: 10.1038/s41419-025-07863-y (PMC12297470; doi:10.1038/s41419-025-07863-y)
Supplement: Supplementary file 3 — qPCR results [file 41419_2025_7863_MOESM3_ESM.pptx]

## Slide 1
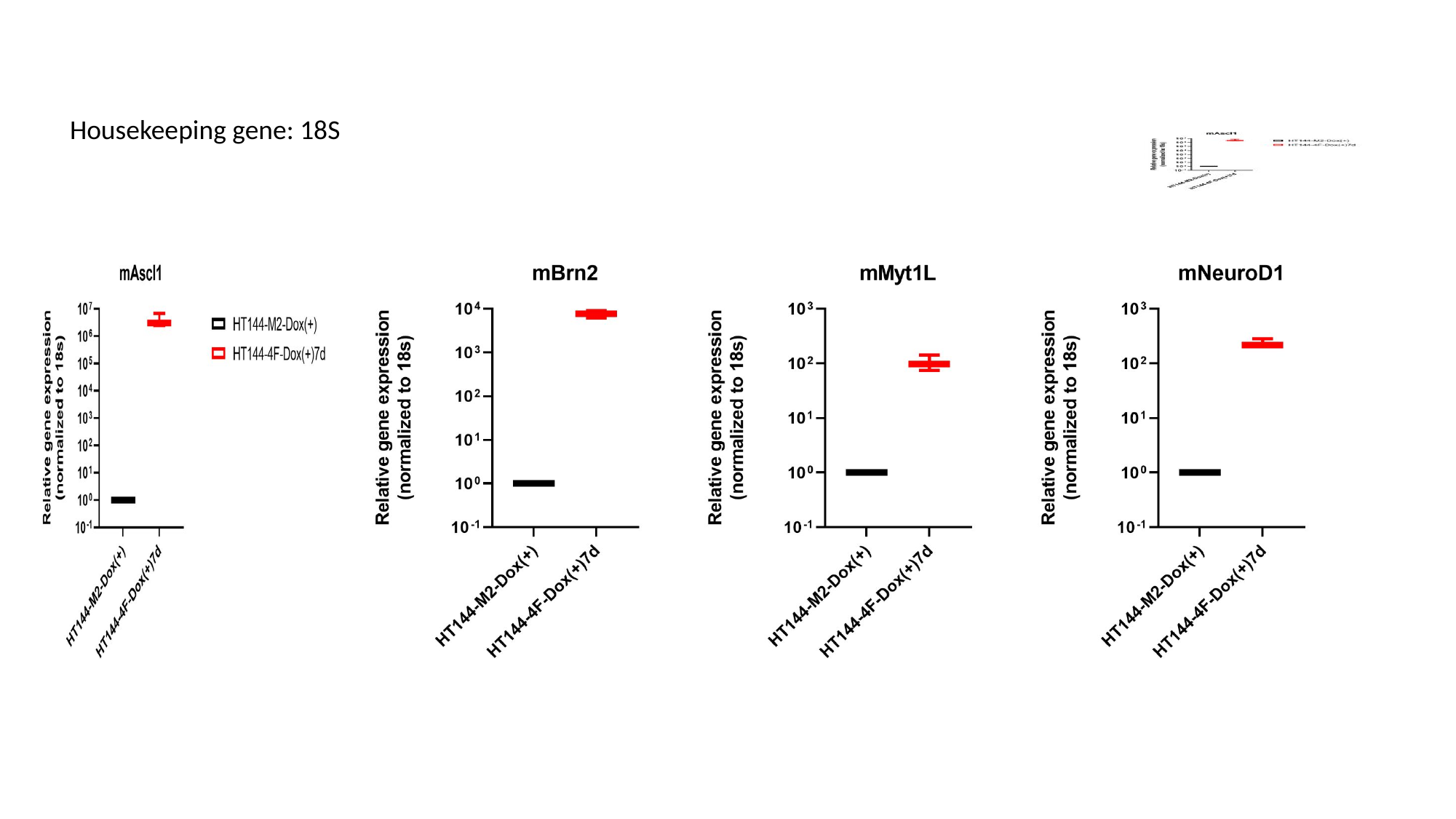

Housekeeping gene: 18S

## Slide 2
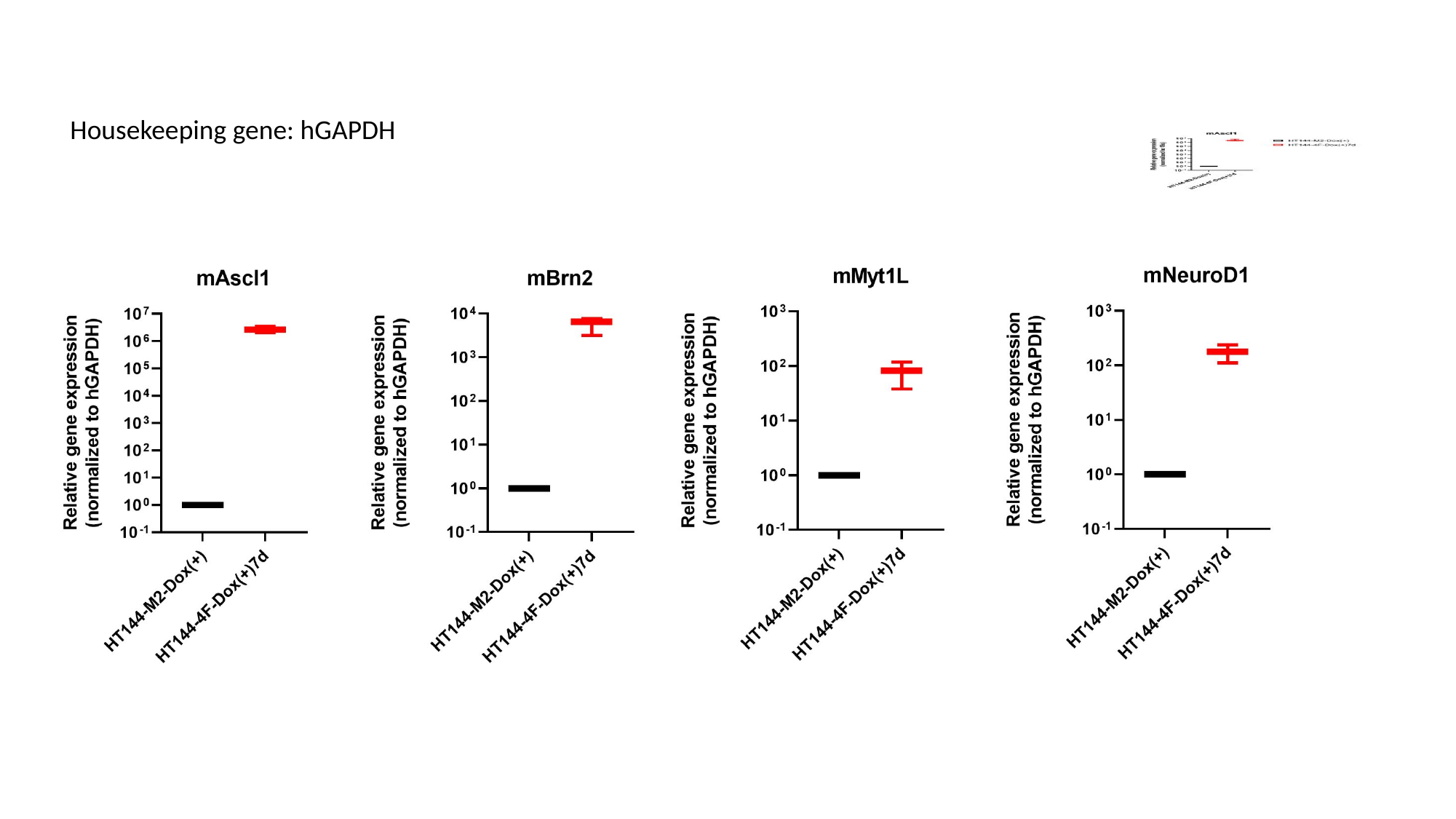

Housekeeping gene: hGAPDH

## Slide 3
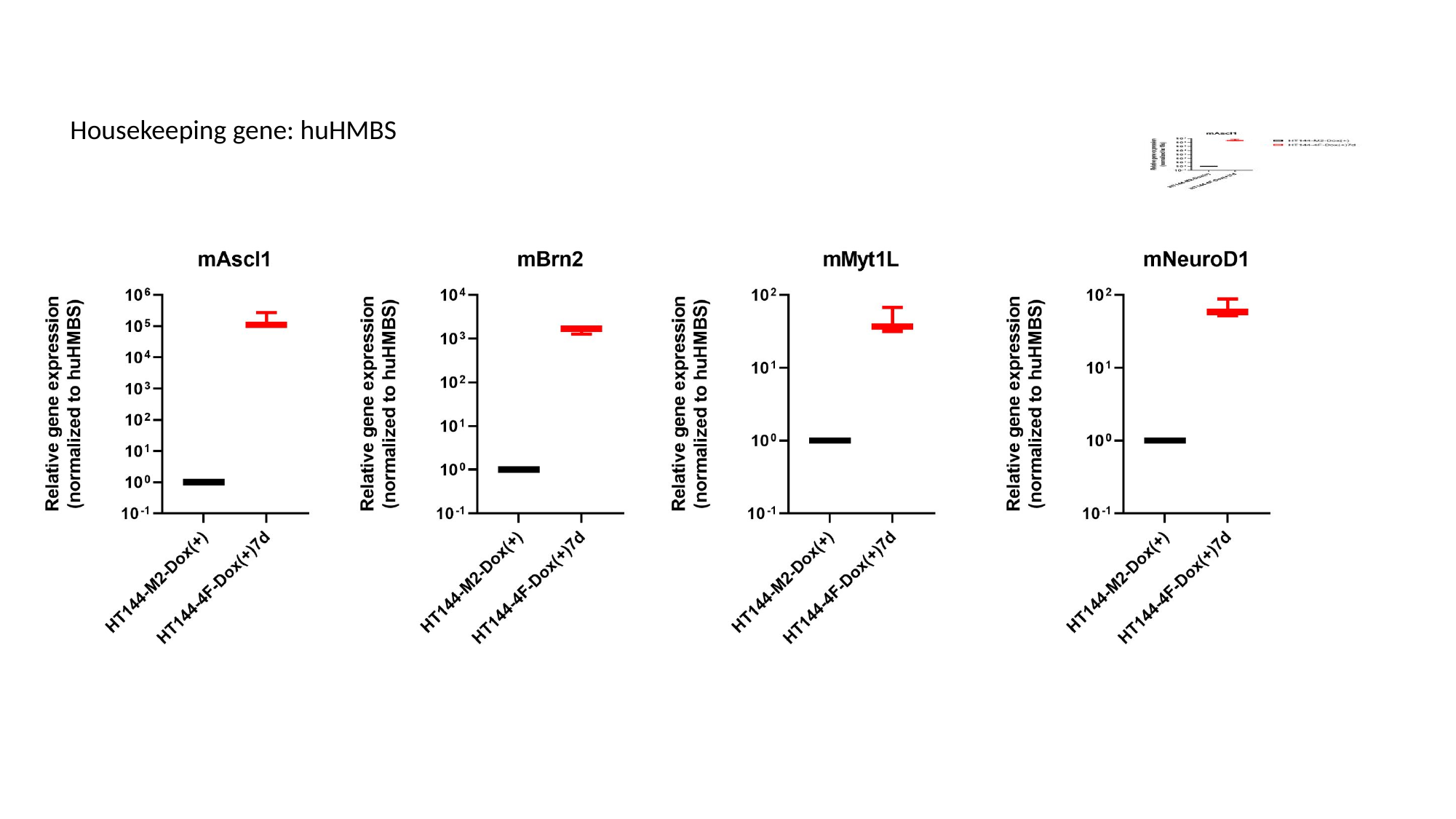

Housekeeping gene: huHMBS

## Slide 4
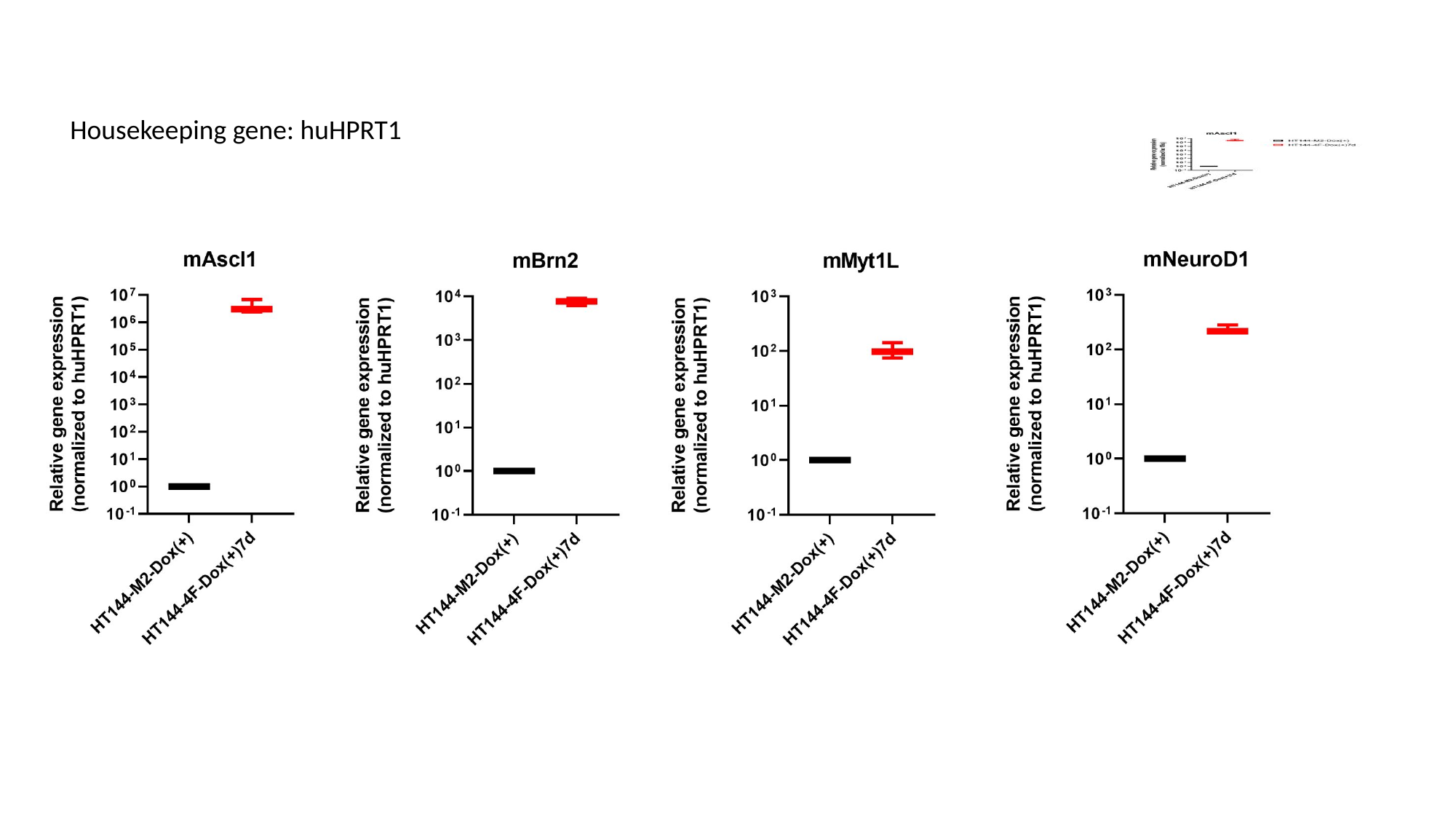

Housekeeping gene: huHPRT1
